# Integrating gestalt therapeutic techniques enhances character portrayal in undergraduate acting students

**DOI:** 10.3389/fpsyg.2026.1697077

**Published:** 2026-03-27

**Authors:** Dian Li, Lu Zhu

**Affiliations:** 1 Acting College, Sichuan University of Media and Communications, Chengdu, China; 2 Graduate School of Business and Advanced Technology Management, Assumption University of Thailand, Bangkok, Thailand

**Keywords:** acting pedagogy, character portrayal, emotional authenticity, gestalt therapy, interdisciplinary integration, performance training

## Abstract

**Introduction:**

Contemporary acting pedagogy in China has long been grounded in the Stanislavsky system to develop character portrayal. However, this traditional approach presents limitations in cultivating students’ emotional accessibility and relational authenticity. This study investigates whether integrating Gestalt Therapeutic Techniques (GTT) with Stanislavski-based training enhances Character Portrayal Proficiency (CPP) in undergraduate acting students.

**Methods:**

A 16-week quasi-experiment compared 71 students in Gestalt + Stanislavski (experimental) and Stanislavski-only (control) groups. Character Portrayal Proficiency was measured across five dimensions. ANCOVA analyzed post-test scores with pre-test covariates. Semi-structured interviews were thematically analyzed in NVivo.

**Results:**

The experimental group significantly improved in four dimensions (Character Analysis, Relational Dynamics, Communication Skills, Internal and External Performance Design), but not Stage Presence. Qualitative data confirmed that Gestalt techniques fostered emotional authenticity, relational engagement, and reflective awareness. Experts acknowledged both pedagogical benefits and implementation challenges, recommending gradual integration of these techniques into acting curricula.

**Discussion:**

The results demonstrate that GTT complements the Stanislavski system by strengthening internal and relational dimensions of performance whilst leaving stage charisma largely dependent on longer-term technical training. These findings affirm the pedagogical value of GTT, suggesting that systematic integration can enrich traditional acting methods and support psychologically grounded actor training.

## 1 Introduction

Contemporary acting pedagogy has long relied on the Stanislavsky system as its foundational paradigm, emphasizing psychological realism and the integration of mind, body, and emotion in performance (McFarren, 2008; Gaurav and Bhardwaj, 2024; Zhang, 2022). Drama education in China has experienced substantial growth, with over 200 university programs dedicated to this field (Zeng, 2019). Although Stanislavsky’s legacy remains foundational, its application has resulted in pedagogical homogenization and a lack of methodological diversity (Zhigang, 2022). This standardization has led to critiques of underdeveloped theatre literacy and limited performance adaptability among graduates (Berry and Brown, 2019; Pitches, 2005).

Recent scholarship suggests that interdisciplinary integration may offer pathways for reform, particularly with psychological methods. Whilst the Stanislavsky system emphasizes inner emotion (Ingleson, 2022). Gestalt therapy is a humanistic approach centered on wholeness, present awareness, and experiential processing (Elliott, 2014; Mann, 2024). Its core concept of ‘experimentation’ (Zinker, 2013), Gestalt therapeutic techniques (GTT)—including the Empty Chair, Dialogue Exercises, Exaggeration, and Role Play, emphasize emotional awareness, present-centeredness, and relational authenticity (Habsy et al., 2024; Josek et al., 2023; Mann, 2020). Though rooted in therapy, these methods align with acting’s core demands, especially in developing Character Portrayal Proficiency (CPP) across emotional, relational, and performative domains (Glickauf-Hughes et al., 1996; Mashkov et al., 2022; Zinn, 2018). Yet GTT remains marginal in curricula, with little empirical research on its educational potential, despite evidence of benefits for resilience, engagement, and relational depth (Avsar and Sevim, 2022; Steyn et al., 2016). Meeting contemporary performance demands requires pedagogy to move beyond static tradition.

Character portrayal training in undergraduate acting education in China continues to face persistent limitations. An over-reliance on traditional methods has marginalized alternative approaches (e.g., Gestalt techniques, psychodrama) and stifled pedagogical innovation (Boeckh, 2014; Fantu, 2024). This leaves many students without the interpretive depth or psychological flexibility demanded by contemporary performance (Berry and Brown, 2019; Clarke et al., 2020). Whilst therapeutic techniques such as Empty Chair and Dialogue Exercises have demonstrated strong efficacy in emotional development and interpersonal learning (Brownell, 2010), they remain largely absent in acting curricula. Conventional training often emphasizes emotional recall and physical action whilst neglecting strategies to manage psychological strain or sustain emotional states in complex roles (Dobreva-Hristova, 2022). Another challenge is the lack of empirical validation for innovative techniques. Gestalt therapeutic tools offer rich potential for improving CPP by fostering emotional authenticity, relational awareness, and embodied expression (Sarasso et al., 2022). However, these techniques have not been systematically tested within acting pedagogy. The absence of standardized evaluation rubrics and controlled research designs has further hindered their academic and institutional recognition (Panero and Winner, 2021). Student engagement also remains a central concern. Repetitive drills and rigid curricula often suppress creativity and intrinsic motivation, leading to disengagement and reduced artistic growth among students (Daud et al., 2024). By contrast, Gestalt-based exercises promote present-centered, emotionally responsive learning that cultivates deeper connection and self-reflection (Holmström et al., 2024). Yet, such techniques are rarely implemented or studied within performance training contexts. Collectively, these challenges highlight the need for a recalibrated pedagogical approach. Integrating GTT with established acting models offers a more holistic, empirically grounded approach to actor training.

Thus, this study aims to explore the pedagogical impact of integrating GTT into undergraduate acting education, with a particular focus on enhancing CPP. Rooted in the recognition that traditional curricula, particularly neglect the psychological and emotional demands of performance (McFarren, 2008), the research responds to calls for more psychologically informed actor training (Chow, 2014). The study specifically investigates how Gestalt specific techniques can be adapted and integrated into the acting teaching to support students’ development across five dimensions of CPP: Character Analysis (CA), Relational Dynamics (RD), Communication Skills (CS), Internal and External Performance Design (IEPD), and Stage Presence (Konopik and Cheung, 2013; Manzanera-Ruiz et al., 2015; Raffagnino, 2019; White, 2023). However, the relationship between specific GTT methods and distinct skill domains in actor training has not been clearly articulated. The following sections introduce a theoretical model that links four key Gestalt techniques to five CPP dimensions. This mapping seeks to clarify how integrate GTT meaningfully to support specific areas of acting student development.

### 1.1 Conceptualising character portrayal proficiency in acting training

CPP refers to an actor’s ability to construct and express a believable character through an integrated combination of emotional authenticity (Garrett, 2024; Alcott, 2024). Also, technical execution and stage presence are essential for character portrayal (Cohen, 2017; Zhang, 2024; Panero and Winner, 2021). As an overarching performance construct, CPP encompasses both the internal preparation and external realisation of a role. In undergraduate actor training, however, its assessment often remains fragmented or overly reliant on subjective impressions, lacking a systematic, multidimensional framework (Goodall, 2008; Jacobs, 2016). To operationalise CPP, this study draws on five interrelated dimensions that together capture the breadth of character portrayal. CA refers to the deep examination of a character’s psychological and emotional dimensions, motivations, and narrative context, integrating both physical and psychological attributes (McFarren, 2008; Mirodan, 2015; Stanley and Strandberg-Long, 2022). Relational Dynamics (RD) highlight the authenticity of interactions between characters, shaped by dialogue, actions, and emotional responses, which are essential for establishing convincing onstage relationships (Ozcelik, 2013; Yang, 2023). Communication Skills (CS) focus on the expressive use of body, face, and voice to convey intentions and emotions, drawing on both verbal and non-verbal techniques to sustain audience engagement (Sun and Okada, 2021; Berry et al., 2022). Internal and External Performance Design (IEPD) concerns the integration of emotional (internal) and physical (external) processes to create coherent and authentic portrayals, emphasising the alignment of inner experience with outward expression (Castilho Barone, 2024; Hodge, 2010). Finally, Stage Presence (SP) refers to the actor’s capacity to command attention and connect with the audience through modulated vocal and physical expressions that are sensitive to spatial and contextual demands (Peppler et al., 2023; Streitberger, 2017).

Taken together, these dimensions offer a multidimensional framework for evaluating CPP, balancing internal authenticity with technical clarity and performance impact. They provide a clear and structured way to assess changes in character portrayal ability. Rather than relying on vague impressions, this approach allows for a focused evaluation of how each skill area is influenced by specific training methods. These dimensions are not isolated; they work together in performance to support a coherent and believable character. This multidimensional view aligns with current perspectives in actor training that stress both psychological depth and technical clarity (Gaurav and Bhardwaj, 2024; Page, 2019).

### 1.2 Gestalt therapy: principles and techniques

Central to the Gestalt perspective is grounded in the concept of “unfinished business,” where unresolved past experiences continue to influence present thoughts and behaviours (Afşaroğlu Eren, 2016; Wagner-Moore, 2004). Gestalt therapy encourages individuals to bring these experiences into conscious awareness and engage with them actively in the “here and now” (García, 2025). In creative and educational settings, these principles can support actors in becoming more aware of their emotions and expressing them with greater authenticity and coherence. This makes GTT particularly relevant for actor training, where accessing emotional truth and embodying complex characters are essential.

Specifically, four core techniques are applied in this study. The Empty Chair Technique involves engaging in self-dialogue with an imagined person or part of the self, increasing awareness and resolving internal conflict (Holmström et al., 2024; Josek et al., 2023; Ndhlovu and Mwanza, 2024). Dialogue Exercises use verbal and non-verbal interaction with parts of the self or imagined others to enhance self-awareness and clarify relational dynamics (Mann, 2020; Paskalieva, 2022; Tucker, 2023). The Exaggeration Technique focuses on amplifying gestures, emotions, or movements to uncover unconscious patterns and strengthen bodily-emotional awareness (Demaree et al., 2004; Hill and Pollick, 2000; Steyn et al., 2016). Role Play requires embodying alternative roles to explore emotional and relational dynamics, fostering empathy and enabling behavioural experimentation (Martinez, 2002). Taken together, these methods are not only therapeutic but also deeply performative, offering structured yet flexible approaches for enhancing expressive depth, emotional access, and character complexity in actor training (Brown, 2006; Brownell, 2010; Trujillo and Holler, 2023).

### 1.3 Pedagogical potential of gestalt techniques in actor training

Recent scholarship has extended Gestalt principles beyond therapeutic settings into higher education pedagogy, demonstrating their potential to foster student agency, creative expression, and community building (Ouzia and Sendra, 2024). Within this broader educational context, GTT align closely with the core aims of acting pedagogy, particularly in fostering emotional authenticity, self-awareness, and relational depth (Passmore and Sinclair, 2020; Polínek, 2016; Vieira and Vandenberghe, 2015). Traditional actor training, especially the Stanislavsky system, emphasizes character analysis, emotional truth, and physical action as routes to compelling performance (Atkins, 2022; Hart, 2020; Stanislavskij, 2021). However, its focus on text and internal psychology can fall short in supporting moment-to-moment responsiveness under performance pressure. GTT offers a practice-based supplement to this model, enabling actors to explore character through structured, experiential processes. Techniques such as the Empty Chair and Dialogue Exercises emphasize on present—centered awareness and relationship realised (Austin and Austin, 2022; Perls et al., 1994). These methods allow actors to externalise internal conflict, explore relational subtext, and practise communication in performance. Role Play and Exaggeration further support the embodied integration of emotion, gesture and presence, key to conveying character through both internal and external design (Mann, 2020; Raindel et al., 2021). Therefore, Gestalt techniques were chosen over other therapeutic approaches (e.g., Morenian Psychodrama) because they align more closely with the moment-to-moment demands of actor training than with psychodrama’s focus on re-enacting past events.

Studies shows that experiential techniques enhance empathy, self-expression, and group cohesion—qualities essential for collaborative rehearsal and performance (Lehtonen et al., 2016; Zelenski et al., 2020). By providing reliable tools for accessing emotion, embodying relational dynamics, and reflecting on personal processes, GTT expands the emotional and psychological dimensions of actor training (Avsar and Sevim, 2022; Stackpole and Quiroga-Garza, 2023). Theoretical exploration further supports this integration, reinforcing its value in bridging therapeutic insight with performance practice.

### 1.4 From theoretical mapping to research objectives

To translate Gestalt therapeutic principles into measurable outcomes in acting training, this research maps four core Gestalt techniques onto the five dimensions of CPP. Whilst several case-based or exploratory studies have applied Gestalt methods in creative education (Andrášik et al., 2024; Holmström et al., 2024; Putri, 2019), their systematic use in actor training remains under-researched. Therefore, the research questions focus:

*Q1*: How do Gestalt therapeutic techniques differentially enhance specific dimensions of character portrayal proficiency in undergraduate acting students?

*Q2*: What are the perceptions of participants regarding the integration of Gestalt therapeutic techniques with traditional acting training?

**Figure 1:**
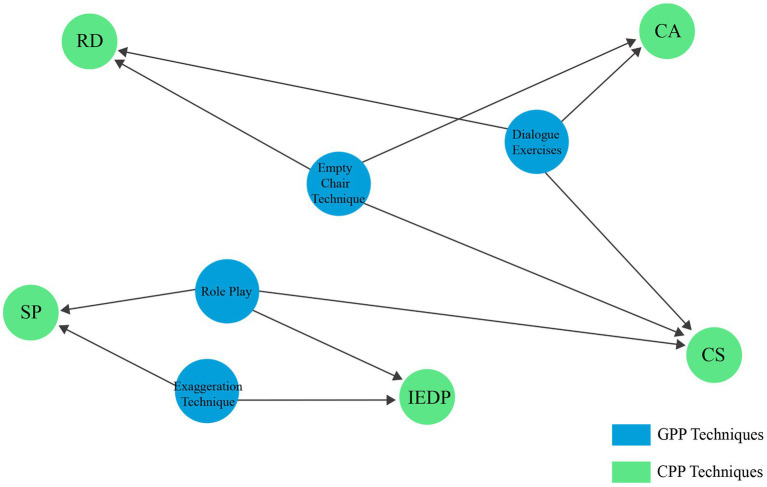
Theoretical design.

The research systematically establishes a functional bridge between psychological process work and actor training outcomes. Each technique targets specific aspects of CPP, offering distinct experiential routes to enhance the actor’s cognitive, emotional, and relational capacities in performance. The Empty Chair Technique, grounded in present-centred self-dialogue, contributes to multiple dimensions of character portrayal. It enhances CA by enabling actors to access unresolved internal conflicts analogous to those of their characters (Fels, 2019). It also strengthens RD and CS by simulating emotionally charged interpersonal scenarios and supports the exploration of authentic emotional responses that translate into embodied character work (Ndhlovu and Mwanza, 2024). Dialogue Exercises, which engage actors in structured inner and outer conversations, deep CA by helping actors articulate a character’s internal conflicts and motivations (Berry and Brown, 2019; Madoyan, 2023). Simultaneously, they strengthen RD and CS by fostering sensitivity to interpersonal nuance and emotional reciprocity (Paskalieva, 2022; Tucker, 2023). Exaggeration, by amplifying physical expression, enhances External Performance Design by refining the actor’s control over gestures, posture, and expressive intensity (Case, 2018). It also strengthens SP by increasing physical clarity and audience impact, helping actors sustain attention through embodied precision and energetic projection (Demaree et al., 2004; Steyn et al., 2016; Hill and Pollick, 2000). Role Play, through immersive enactment of imagined scenarios, enhances CS by encouraging spontaneous verbal and non-verbal expression, sharpens IEPD by aligning inner emotional states with physical embodiment, and cultivates SP by fostering confidence and sustained audience connection (Pugh et al., 2001; Mirodan, 2015; Nair, 2019). These mappings are visually summarised in the theoretical diagram (see Figure 1), which illustrates the dynamic interrelation between GTT methods and the core components of CPP.

This study hypothesises that undergraduate acting students receiving GTT-based training will show significantly greater improvement across all five CPP dimensions—CA (H1), RD (H2), CS (H3), IEPD (H4), and SP (H5)—than those taught exclusively through Stanislavsky techniques. Accordingly, the theoretical design is addressed in the following research objectives.

1. To evaluate whether the integration of GTT leads to significant improvements in CPP and its five component skills.
2. To assess participants’ attitudes towards the integration of Gestalt therapeutic techniques with traditional Stanislavsky methods.

The significance of this research lies in its interdisciplinary approach and empirical orientation. By aligning these techniques with performance training objectives, the study offers a novel framework and practical evidence for enriching acting curricula, particularly in the Chinese context where methodological plurality is lacking. Beyond its methodological contributions, the research also addresses issues of student engagement and emotional authenticity in performance. Gestalt-based practices, by contrast, emphasize presence, spontaneity, and emotional truth—qualities that resonate strongly with the demands of contemporary theatrical teaching.

## 2 Methods

### 2.1 Study design

**Figure 2:**
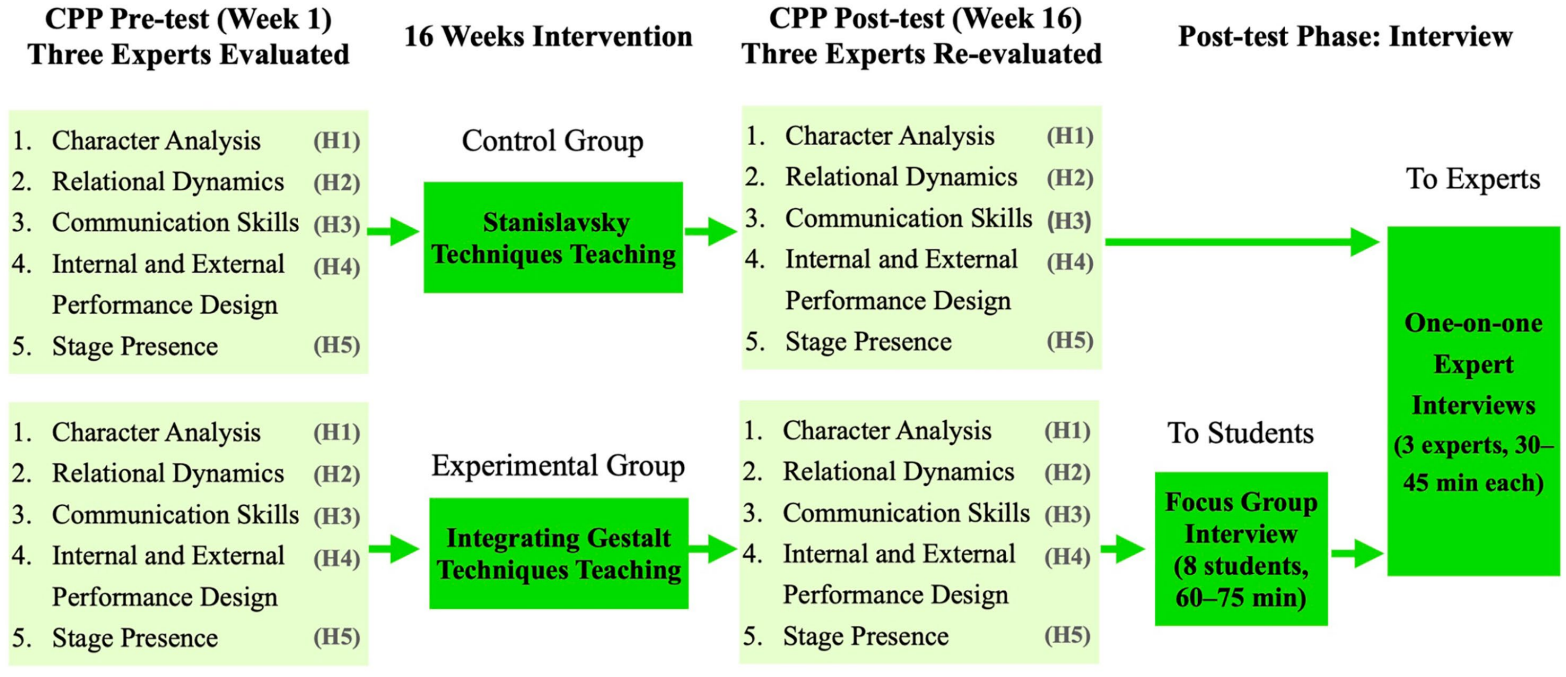
Study design.

The mixed-methods design (Figure 2) to evaluate the pedagogical impact of GTT integrated with Stanislavsky methods on undergraduate acting students’ CPP. The approach combines quantitative and qualitative data to investigate both performance outcomes and participant experiences. Quantitatively, the research follows a pre-test/post-test experimental design with two groups: an experimental group receiving integrated GTT-Stanislavsky training, and a control group taught solely through Stanislavsky-based methods. CPP is assessed across five dimensions: CA, RD, CS, IEPD, and SP. These dimensions reflect the official CPP evaluation scale developed by Sichuan University of Media and Communications (2023). Pre-tests were conducted in Week 1, with post-tests held in Week 16, both scored by three independent acting experts of SUMC. The intervention lasted 16 weeks, consistent with SUMC’s semester structure and pedagogical objectives. This duration was considered appropriate as Gestalt techniques do not follow a fixed cycle and can be effectively implemented within flexible periods, as shown in a two-month case study by Aiach Dominitz (2017). Moreover, CPP development requires an extended rehearsal process; Snyder-Young (2011) reported a 12-week rehearsal cycle for stage performance. A 16-week design therefore allowed adequate time for both the delivery of GTT interventions and the consolidation of CPP. Analysis of Covariance (ANCOVA) was used to analyse post-test data, controlling for baseline variation via pre-test scores as covariates. This ensured a robust comparison of performance improvements across groups. Qualitatively, two forms of interviews were conducted. First, semi-structured interviews with the three experienced assessors after 16 weeks explored their evaluations of GTT’s pedagogical contributions. Second, eight students joined the focus group interview. This group size was consistent with methodological guidance for focus group research, which recommends 6 to 12 participants to support interactive yet in-depth discussion (Sagoe, 2015). Interviews were conducted after the post-test to capture students’ reflections on their experiences with Gestalt therapeutic techniques. Thus, the focus group interview was captured experiential insights into the use and effectiveness of GTT. Both sets of qualitative data were thematically analysed using NVivo 15.

### 2.2 Participants and ethical considerations

According to official figures from the Sichuan University of Media and Communications (2023), the 2023 undergraduate acting cohort comprised 355 students across ten classes. From this cohort, two parallel classes were purposively selected: Class 03 (experimental group, *n* = 36) and Class 05 (control group, *n* = 35). Both classes had comparable gender distributions (Class 03: 18 females, 50.0%; Class 05: 18 females, 51.4%) and were assigned through the same entrance examination, ensuring baseline equivalence. In total, 71 students participated, all of whom performed in *The Last Empress and Der Ling* under a shared rehearsal schedule, providing consistent training conditions. To calculate the sample size, the G*Power 3.1 software is utilised for sample size estimation. The G*Power software is designed to provide an accurate power analyses based for most statistical tests in behavioural science (Faul et al., 2007). In this study, *a priori* test (*f* = 0.35) indicated that 67 participants were required; with 71 available, this threshold was exceeded. Sensitivity analysis showed that N = 71 could detect effects of *f* ≥ 0.34 (η^2^ ≈ 0.10). A *post hoc* test confirmed achieved power above 0.82. Following Cohen’s (2009) benchmarks, f = 0.35 falls between a medium (0.25) and a large (0.40) effect, underscoring the adequacy of the sample size. Demographic comparability across gender, age, and regional background further supported the study’s internal validity. Most participants were born in 2004, consistent with the expected age of undergraduate students. In addition, their distribution across all seven regions of China shows that the cohort represented a broad geographical background. Together, these demographic features demonstrate that the sample was both typical and diverse, reinforcing the accuracy and wider significance of the experiment. The choice of second-year students was also pedagogically appropriate: by this stage they had completed foundational first-year training, which provided the basic skills necessary for CPP pre-testing. Moreover, within the SUMC (2024–2025) curriculum, the instructional goal for second-year acting majors is to achieve an initial level of CPP, thereby laying the foundation for the more advanced demands of full stage productions in the third year.

Ethical approval was obtained from the Institutional Review Board at Assumption University of Thailand. Informed consent was collected from all student participants using bilingual forms (Chinese and English) outlining the study’s aims, procedures, potential risks, benefits, and rights, including voluntary participation and data confidentiality. Anonymization and secure data storage were strictly maintained. In addition, the group of experts comprised three senior evaluators, who independently assessed CPP during pre-test and post-test performances. Three experts with distinguished professional backgrounds served as evaluators of CPP. Expert 1 is a National First-Class Actress and recipient of two Golden Rooster Awards. Expert 2 is a Professor and recognised authority on national acting examination standards. Expert 3 is a Professor, accomplished director and actor with over 100 notable works. Their extensive expertise ensured the credibility and rigour of the CPP assessment. These three experienced evaluators participated under the same ethical principles, conducting assessments based on the university’s official performance criteria, thereby ensuring autonomy, beneficence, non-maleficence, and justice throughout the research process.

### 2.3 Instruments

**Figure 3:**
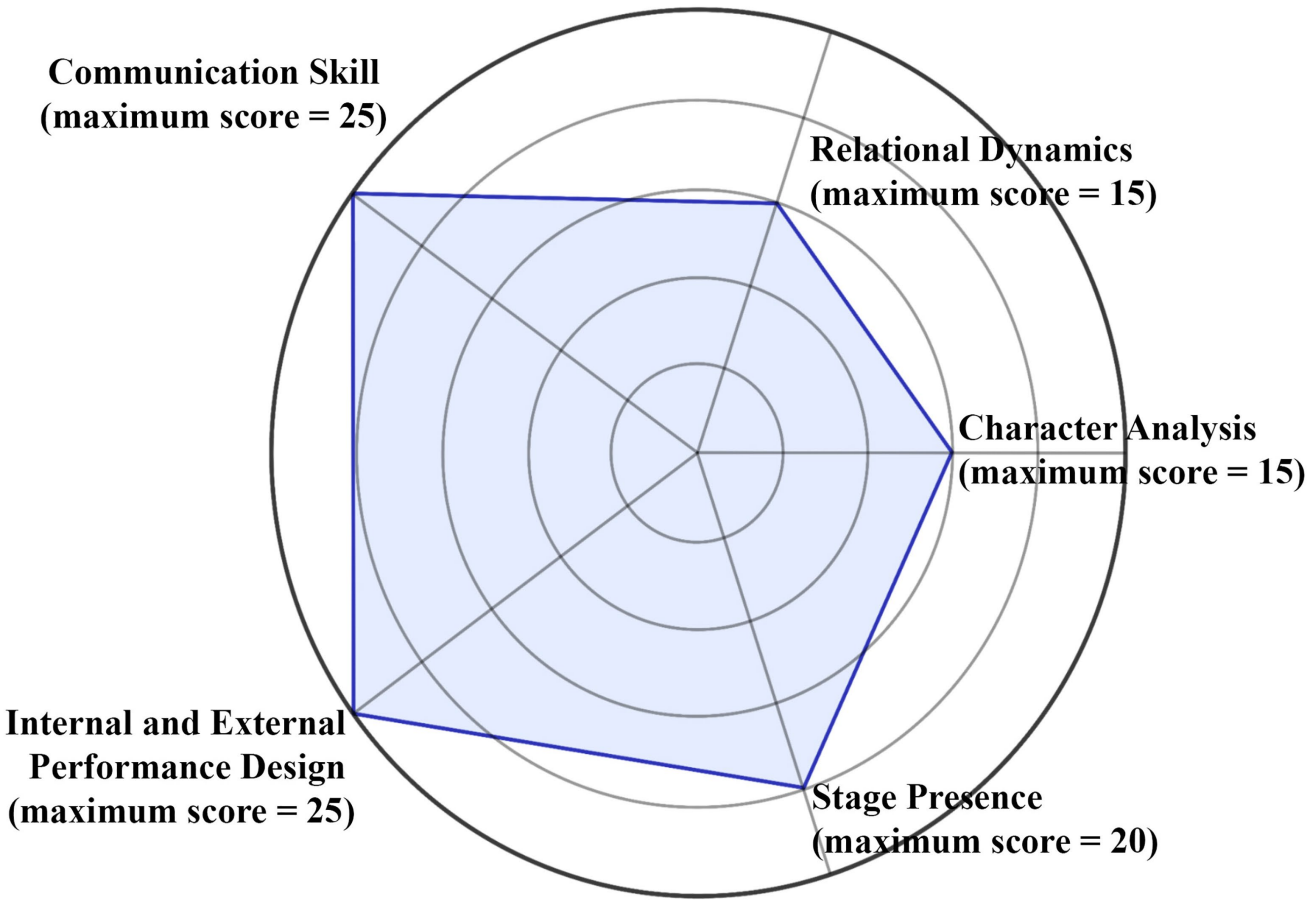
CPP assessment dimensions and weight distribution (total 100 score).

This study employed two primary instruments: a performance-based assessment of CPP and a set of semi-structured interview protocols. The CPP test was adapted from the official assessment scale and evaluated across five weighted dimensions (CA 15%, RD 15%, CS 25%, IEPD 25%, SP 20%), as illustrated in Figure 3. CA was assessed through a written report, whilst the remaining dimensions were judged through live performance. Three experienced evaluators independently scored each assessment. They were briefed to ensure consistent application of the rubric, which was grounded in the university’s established performance criteria (Table 1).

**Table 1:** Character portrayal proficiency assessment criteria (Sichuan University of Media and Communications, 2025).

Assessment item	Scale guidelines	Treatment	Weight
Character analysis	11–15 points: provides an in-depth understanding of the character’s motivations, background, and context. 6–10 points: demonstrates partial understanding with general insights. 0–5 points: displays limited understanding, lacks depth or clarity.	Submission of a character analysis report	15%
Relational dynamics	11–15 points: accurately and convincingly portrays relationships with other characters. 6–10 points: shows general ability to depict relationships but lacks depth or consistency. 0–5 points: portrayal of relationships is unclear or lacks authenticity.	Performance test	15%
Communication skills	20–25 points: demonstrates highly effective verbal and non-verbal communication, including clear articulation, appropriate gestures, and expressive body language. 10–19 points: communication is moderately effective, with some inconsistencies in delivery or expression. 0–9 points: ineffective communication, lacking clarity, expressiveness, or appropriate non-verbal cues.	Performance test	25%
Internal and external performance design	20–25 points: successfully integrates emotional depth with physical movement, creating a cohesive and engaging performance. 10–19 points: shows partial integration of emotional and physical performance, with room for improvement. 0–9 points: limited connection between emotional expression and physical movement, leading to a disjointed performance.	Performance test	25%
Stage presence	15–20 points: presents a complete and polished performance with clear rhythm and strong audience impact. 8–14 points: presents a mostly complete performance, but rhythm and engagement are inconsistent. 0–7 points: performance lacks completion, rhythm, and audience engagement.	Performance test	20%

The CPP assessment scale (Table 1) provides detailed scoring guidelines across the five dimensions. Each dimension is divided into three performance bands, with descriptors that distinguish high, moderate, and low achievement. For example, high-level CA reflects a clear grasp of character motivation and context, whilst low CA shows minimal depth or clarity. Strong RD is marked by authentic and convincing interactions, whereas weak RD lacks consistency or authenticity. High CS requires clarity in both verbal delivery and nonverbal expression, whilst low CS reflects ineffective or unclear communication. In IEPD, top scores integrate emotional depth with physical movement to form a cohesive performance, whilst weak performances show disconnection between the two. Finally, strong SP combines rhythm and audience impact, whilst weak SP is characterised by inconsistency and disengagement. The weighted structure (CA 15%, RD 15%, CS 25%, IEPD 25%, SP 20%) ensured balanced evaluation across cognitive and performative aspects of training.

To complement quantitative outcomes, two interview protocols were developed: a focus group for students (n = 8) and individual semi-structured interviews for the three experienced evaluators. Question routes were mapped to the five CPP dimensions and to the Gestalt techniques implemented during training. Instruments were bilingual and underwent Item-Objective Congruence (IOC) review by external experts in psychology and drama education. All interviews were conducted post-intervention, audio-recorded with consent, and transcribed verbatim for thematic analysis. The student group size is consistent with recognised focus-group. Table 2 summarises the focal domains for each group.

**Table 2:** Overview of interview focus areas for students and experts.

Target group	Focus areas	Illustrative focus
Students	CA	Exploring how GTT supports understanding of character motivation, internal conflict, and role interpretation.
(Focus group interviews)	RD	Examining how GTT fosters believable on-stage relationships and emotional interaction.
	CS	Investigating the role of GTT dialogue and exercises in enhancing verbal and non-verbal expression.
	IEPD	Considering how GTT techniques link emotional depth with physical embodiment and expressive movement.
	SP & confidence	Assessing how GTT contributes to authenticity in climactic scenes and strengthens performers’ confidence and stage presence.
	Evaluation of CPP dimensions	Assessing the influence of GTT across the five dimensions of CPP.
(Semi-structured interviews)	Pedagogical effectiveness of GTT	Comparing GTT with Stanislavsky-based training and identifying unique contributions.
	Observed challenges	Identifying practical limitations, implementation difficulties, or technical concerns in applying GTT.
	Long-term value & recommendations	Exploring the sustainability of GTT in actor training and future directions for pedagogy and research.

CA, Character Analysis; RD, Relational Dynamics; CS, Communication Skills; IEPD, Internal and External Performance Design; SP, Stage Presence.

Content validity for the qualitative instruments was addressed via IOC review alongside bilingual preparation. For the CPP assessment, validity is anchored in the university’s officially adopted evaluation criteria and their long-standing departmental use. Reliability was supported through (i) independent scoring by three senior experienced evaluators at both pre- and post-test, and (ii) prior briefing to standardise application of the rubric. These procedures, together with the explicit scoring descriptors and weights in Table 1, were designed to minimise subjectivity and enhance inter-rater consistency. The research instruments were professionally translated to ensure accuracy and reliability, with the full translated versions provided in the Supplementary material.

### 2.4 Procedure

This study followed a 16-week experimental design involving two intact groups of undergraduate acting students. In Week 1, both the experimental and control group completed a baseline performance assessment using the university’s official CPP rubric. Each student delivered a short oral presentation for CA Test and participated in small-group rehearsals of selected scenes. Performances were assessed by three experts. During Weeks 2–15, the experimental group received integrated instruction combining GTT with Stanislavsky-based training, whilst the control group followed the standard Stanislavsky curriculum. In Week 16, both groups presented final performances of *The Last Empress and Der Ling*, including CA written work, which were re-evaluated by a panel of three experts. This structure enabled a comparative analysis of the impact of GTT across five CPP dimensions. Both groups followed a 180-min weekly session. The control group received a traditional Stanislavsky-based curriculum, including warm-ups, theory, exercises, and scene rehearsals. The experimental group followed the same structure but dedicated 30 min each week to GTT, directly mapped onto CPP dimensions (Details in Table 3). GTT activities were practiced in small groups of two to three students in experimental group. The instructor gave prompts and demonstrations, after which one student guided peers through the exercise in relation to their character. Roles were then rotated to ensure all students experienced both guiding and enactment.

**Table 3:** Weekly session structure for control and experimental groups.

Stage	Control group (Stanislavsky-based)	Experimental group (Stanislavsky + GTT)	Time allocation
1. Introduction & Ice-break	Course overview, expectations, and short ice-break activity	Course overview, goals, introduction to GTT, and ice-break activity	10 min
2. Warm-up	Physical and vocal warm-ups (stretching, breathing, articulation)	Physical and emotional warm-ups with partner-based focus exercises	15 min
3. Theories of CPP	Overview of CPP dimensions and examples from performances	CPP dimensions with examples from *The Last Empress and Der Ling*, discussion of GTT relevance	20 min
4. Stanislavsky methods	Emotional memory, subtext, physical actions; guided feedback	Emotional memory, subtext, physical actions; emphasis on linking to GTT	30–45 min
5. GTT	—	30 min: Empty Chair, Dialogue Exercises, Role Play, Exaggeration (applied to CPP dimensions)	30 min
6. Presence & scene work	Scene rehearsals focusing on stage presence, emotional connection, ensemble work	Scene rehearsals with GTT integration, refining emotional–physical connections, ensemble dynamics	70–85 min
7. Conclusion & homework	Summary of session, reflective homework on character analysis	Summary of session, reflective homework on how GTT supported performance	5 min

**Figure 4:**
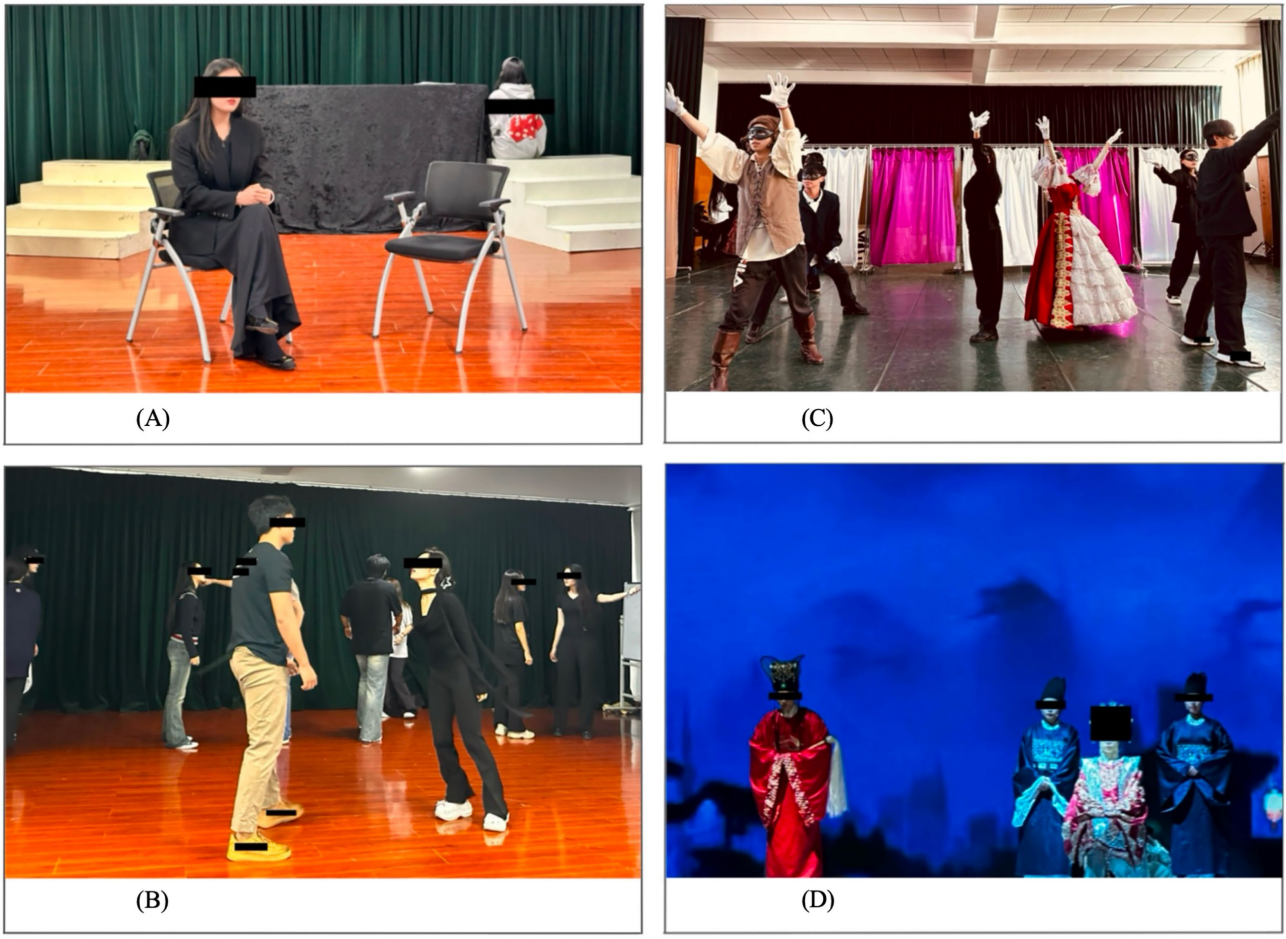
Applications of gestalt techniques in actor training. **(A)** Empty chair technique during rehearsal. **(B)** Dialogue exercises during rehearsal. **(C)** Exaggeration technique during rehearsal. **(D)** Role play during the final rehearsal.

Each technique was aligned with core acting competencies to support cognitive, emotional, and physical embodiment. The integration of GTT followed the sequence of the university’s CPP training plan, with each method aligned to the weekly focus of character portrayal. From Weeks 2 to 8, the Empty Chair Technique was introduced to strengthen emotional authenticity and character motivation. Students engaged in internal dialogues by projecting imagined figures of *The Last Empress and Der Ling* into an empty chair to explore conflicting ego states (Holmström et al., 2024; Staemmler, 2018; White, 2023). This exercise deepened psychological engagement and clarified relational intent, directly supporting the development of CA, RD, and CS. As illustrated in Figure 4A, a photo of a student in the experimental group practicing the Empty Chair Technique during rehearsal. In this exercise, the student assumed the role of her character whilst imagining her younger self seated in the opposite chair. The dialogue with the imagined figure facilitated deeper CA, enhanced awareness of RD, and generated authentic communication of CS, thereby supporting the development of monologue performance.

Alongside this, Dialogue Exercises (Weeks 2–8) encouraged spontaneous, emotionally grounded exchanges with imagined figures from the characters’ backstories. Confirmation was encouraged through structured turn-taking, where each student was explicitly tasked with acknowledging their partner’s emotional offering before responding. Inclusion was fostered by prompting students to momentarily adopt their partner’s perspective, deepening empathic engagement whilst maintaining their own boundaries. By emphasizing present-centered awareness and improvisational responsiveness, these exercises further reinforced CA, RD, and CS (Garcia, 2024; Mäyrä, 2017). Figure 4B illustrated students practiced the Dialogue Exercises in small groups, immersing themselves in their roles whilst engaging in sustained exchanges with one another. Each student aimed to be understood and responded to within the interaction, creating a heightened sense of relational authenticity. Unlike the traditional Gestalt format, this adapted form of Dialogue Exercises was interwoven with rehearsal objectives, making it more suitable for actor training and performance preparation. By immersing themselves in their roles and engaging in sustained exchanges, students developed deeper character analysis (CA), strengthened relational dynamics (RD), and enhanced communication skills (CS).

From Weeks 9 to 15, the Exaggeration Technique was employed to heighten external expressiveness. Students amplified habitual gestures, vocal patterns, or movements to uncover unconscious emotional material, which was then re-integrated into controlled and stylized performance choices (Habsy et al., 2024; Mäkäräinen et al., 2014). This process supported IEPD and SP by linking inner states to external form and by strengthening clarity, projection, and audience impact. Figure 4C depicts students practiced the Exaggeration Technique by magnifying bodily postures to their extremes. Props such as masks and gloves were incorporated, shifting the focus away from realistic representation and towards an exploration of symbolic expression. The exercise emphasized symbolic over realistic representation, strengthening internal and external performance design (IEPD) and stage presence (SP). This approach exemplified how the Exaggeration Technique can be creatively adapted for actor training, highlighting its value in connecting physical exaggeration with pedagogical experimentation.

From Weeks 5 to 15, Role Play was used to extend learning in CS, IEPD, and SP. Through unscripted and emotionally charged scenarios, students explored a broader behavioural range and refined performance choices in real time (Martinez, 2002). Solo and partner improvisations included role reversals and enactments of daily habits and movement patterns, helping students embody characters’ emotional and physical responses more fully (Macfie et al., 2005; Yaniv, 2012). Figure 4D, taken during the final rehearsal in Week 15, shows students in full costume and makeup engaging in Role Play. Students demonstrated physical actions and effective character embodiment, reflecting the pedagogical aims of the training in communication skills (CS), internal and external performance design (IEPD), and stage presence (SP). All identifiable individuals in these images provided separate written informed consent for the publication of their photographs.

To complement the quantitative data, a focus group interview was conducted with eight students from the experimental group, selected through stratified random sampling by gender and baseline CPP tertiles. From the 36 eligible students in the experimental group, eight were randomly chosen within strata using a computer-generated sequence, with same-stratum replacements applied in cases of refusal. The session, held in the final week after the stage performance, lasted 60–75 min, a duration consistent with recommended practice for focus groups, which typically run 60–90 min to balance depth and engagement (Nuttavuthisit, 2019). In addition, semi-structured interviews were held with the experienced evaluators once the intervention concluded, allowing them to provide holistic assessments of the training. Each lasted 30–45 min, aligning with common practice for qualitative interviews (Britten, 2014), and explored their assessments of GTT’s impact, challenges observed, and its potential integration into curricula. All interviews were audio-recorded, transcribed, translated, and proofread prior to thematic coding and analysis in NVivo 15. These interviews captured both student and evaluator perspectives on the integration of GTT into actor training.

### 2.5 Data analysis

The quantitative data were analysed using SPSS Statistics 30, whilst qualitative data from interviews were processed through NVivo 15, ensuring a comprehensive interpretation of both performance outcomes and participant experiences. The primary statistical method employed was ANCOVA, used to determine post-test differences in CPP between the experimental and control groups whilst controlling for pre-test scores. This method was chosen to account for baseline variability and to isolate the effects of the GTT-based intervention. Five separate ANCOVA tests were conducted for each CPP dimension. A significance threshold of *p* < 0.05 was applied across all tests. Descriptive statistics (means, standard deviations, and frequency distributions) were also computed to describe group characteristics and performance trends across the pre-test and post-test phases. To complement the quantitative findings, thematic analysis was conducted on two sets of interview data. NVivo 15 was used to code transcripts line-by-line and cluster codes into broader themes aligned with the five CPP dimensions and the four GTT techniques. Cross-case comparison and frequency analysis were used to identify consistent patterns and emergent categories with quantitative outcomes. Coding frameworks were developed deductively from the study’s theoretical model and refined inductively during iterative analysis, following the guidance of Patton (2015).

## 3 Results

### 3.1 Descriptive statistics

**Figure 5:**
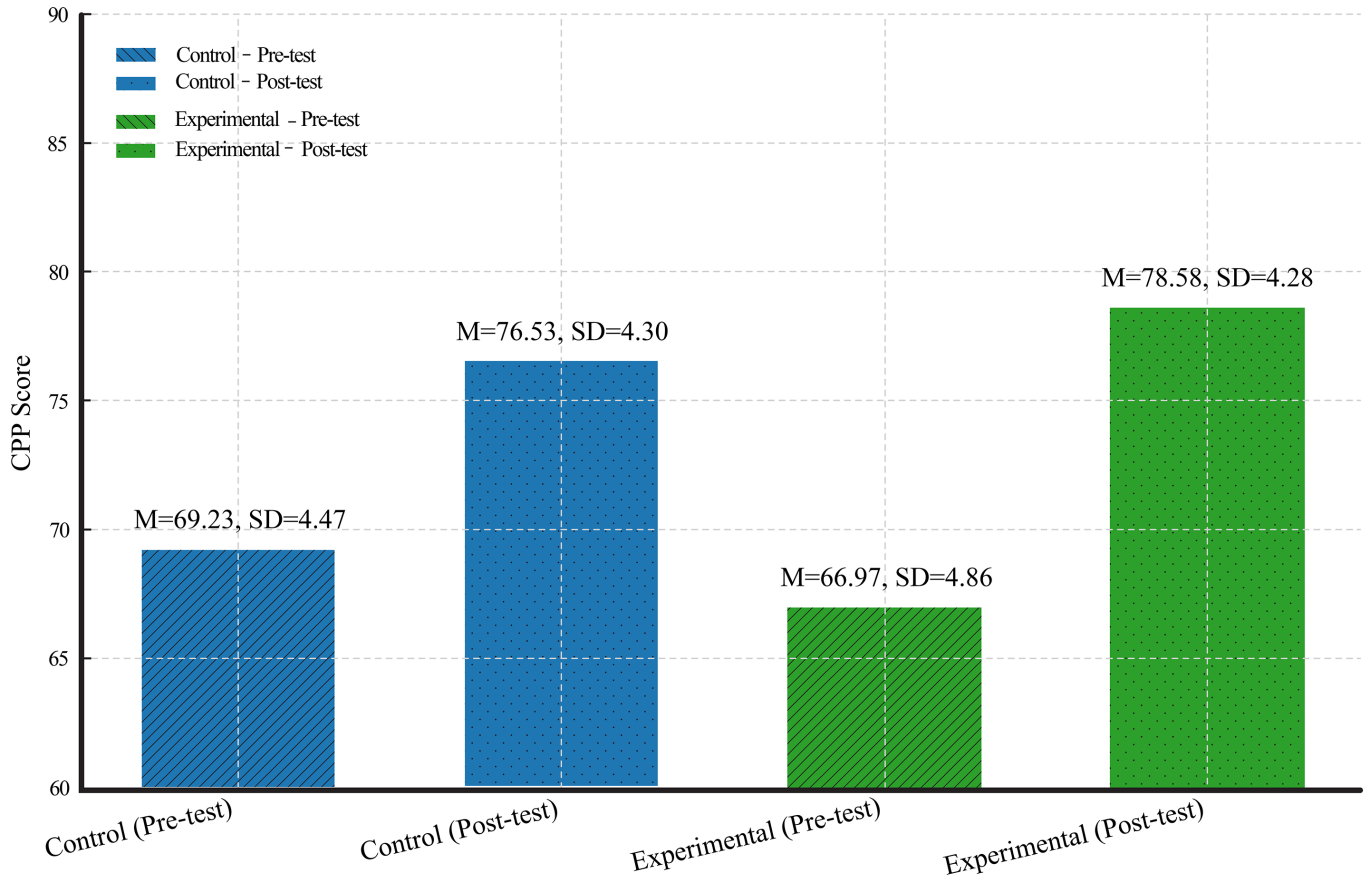
Descriptive statistics of CPP scores by group.

Descriptive statistics were calculated to establish a baseline for the two groups and to examine overall trends in CPP development. As shown in Figure 5, both the experimental and control groups recorded gains in their total CPP scores from pre-test to post-test. At pre-test, the control group had a mean total CPP score of 69.23 (SD = 4.47), with scores ranging from 62 to 83, whilst the experimental group started with a slightly lower mean of 66.97 (SD = 4.86), ranging from 57 to 81. Following the 16-week intervention, both groups showed improvement. The control group’s mean post-test score increased to 76.53 (SD = 4.30), with a range of 64 to 88, representing a 7.3-point gain. In comparison, the experimental group’s mean post-test score rose more substantially to 78.58 (SD = 4.28), with a range of 66 to 90, reflecting an 11.6-point increase from baseline.

**Figure 6:**
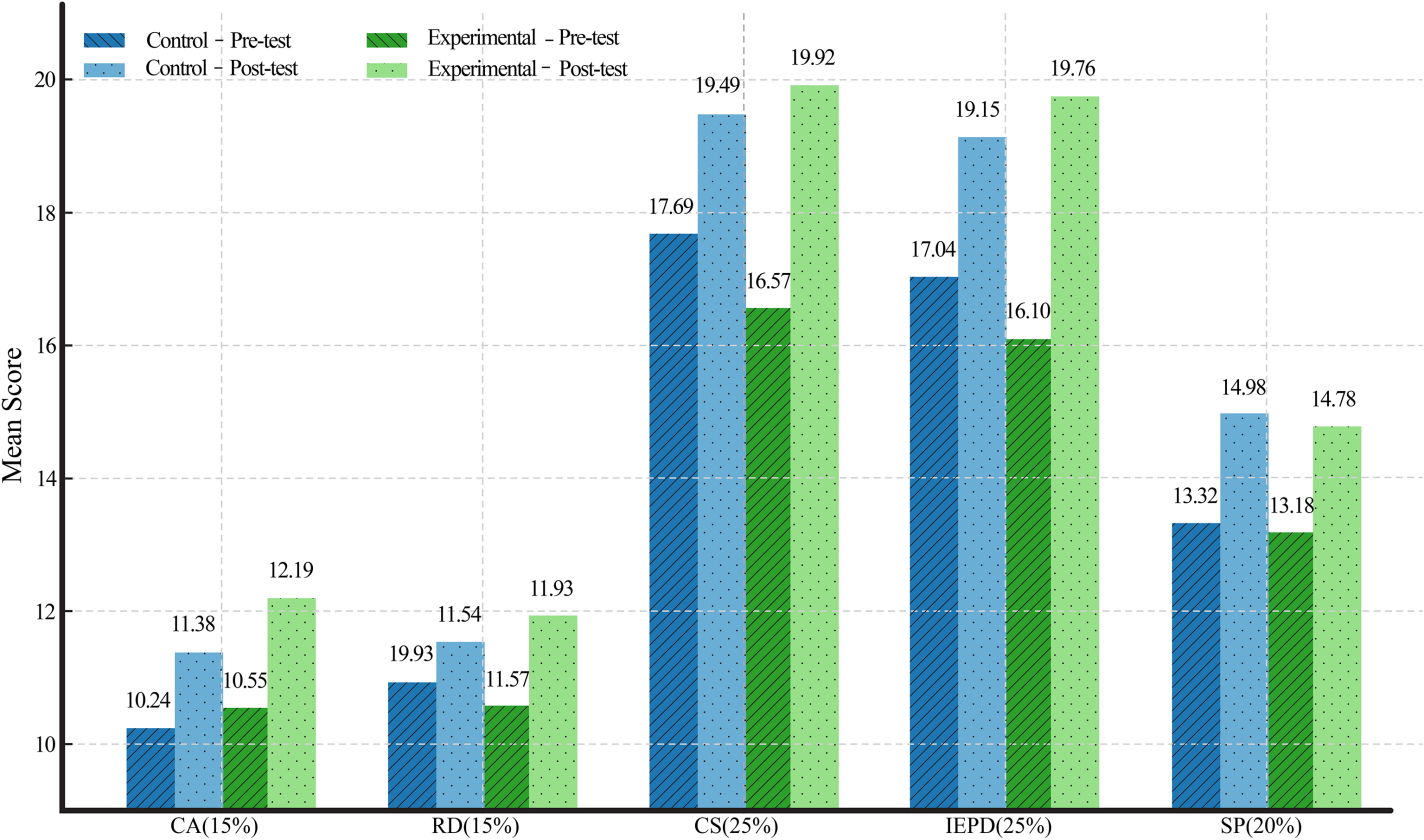
Mean CPP scores by dimension by group.

To explore how this improvement was distributed across different dimensions of performance, descriptive data were also examined for each of the five CPP dimensions. Across individual CPP dimensions, both groups showed upward trends in mean scores. Notably, in the experimental group, the most pronounced gains were observed in CA (from *M* = 10.55 to *M* = 12.19), IEPD (from *M* = 16.10 to *M* = 19.76), and CS (from M = 16.57 to *M* = 19.92). Similarly, the control group showed improvements in the same areas, albeit to a lesser extent. Visual comparisons further illustrate these patterns. Figure 6 displays the overall progression dimension scores across both groups, showing steeper gains in the experimental group.

### 3.2 Quantitative phase

#### 3.2.1 ANCOVA results

Prior to conducting ANCOVA (Table 4), the homogeneity of regression slopes assumption was tested by examining the interaction between group membership and pre-test scores for each dependent variable. None of the interaction effects reached significance (*p* = 0.106–0.710), confirming that the assumption was met and ANCOVA could be applied to adjust for baseline differences.

**Table 4:** Tests of homogeneity of regression slopes for CPP dimensions.

Source	Type III sum of squares	Mean square	*F*	*P*	Partial eta squared
Group *CA (PRE)	0.399	0.399	1.663	0.202	0.024
Group * RD (PRE)	0.333	0.333	2.683	0.106	0.038
Group * CS (PRE)	0.545	0.545	1.013	0.318	0.015
Group * IEPD (PRE)	0.944	0.944	1.197	0.278	0.018
Group * SP (PRE)	0.111	0.111	0.14	0.710	0.002
Group * Total Score (PRE)	2.379	2.379	0.322	0.572	0.005

CA, Character Analysis; RD, Relational Dynamics; CS, Communication Skills; IEPD, Internal and External Performance Design; SP, Stage Presence.

After controlling for pre-test scores, significant group effects were found in four of the five CPP dimensions and the total score, which are summarised in Table 5. For CA, the experimental group outperformed the control group, *F* (1, 67) = 30.856, *p* < 0.001, partial *η*^2^ = 0.312, indicating a moderate-to-large effect. RD showed the largest effect size, *F* (1, 67) = 42.303, *p* < 0.001, partial *η*^2^ = 0.384, reflecting notable gains in the ability to sustain believable onstage relationships. CS also differed significantly between groups, *F* (1, 67) = 41.535, *p* < 0.001, partial *η*^2^ = 0.379, with the experimental group demonstrating clearer articulation and stronger expressive responsiveness. For IEPD, the group effect was significant, *F* (1, 67) = 27.006, *p* < 0.001, partial *η*^2^ = 0.284, suggesting greater integration of psychological intention and physical execution among experimental participants. By contrast, SP did not differ significantly, *F* (1, 67) = 0.181, *p* = 0.672, partial *η*^2^ = 0.003, indicating that improvements in this area were similar in both groups. The ANCOVA on total CPP score confirmed a significant advantage for the experimental group, *F* (1, 67) = 30.895, *p* < 0.001, partial *η*^2^ = 0.312, supporting the overall effectiveness of the intervention.

**Table 5:** ANCOVA results for post-test scores with pre-test scores as covariates.

Items	Source	Type III sum of squares	*F*	Sig.	Partial eta squared
CA	CA (PRE)	15.521	64.023	<0.001	0.485
	Group	7.48	30.856	<0.001	0.312
RD	RD (PRE)	12.997	102.296	<0.001	0.601
	Group	5.375	42.303	<0.001	0.384
CS	CS (PRE)	68.196	126.682	<0.001	0.651
	Group	22.359	41.535	<0.001	0.379
IEPD	IEPD (PRE)	41.703	52.759	<0.001	0.437
	Group	21.346	27.006	<0.001	0.284
SP	SP (PRE)	41.412	52.88	<0.001	0.437
	Group	0.142	0.181	0.672	0.003
Total Score	Total Score (PRE)	773.39	105.852	<0.001	0.609
	Group	225.73	30.895	<0.001	0.312

CA, Character Analysis; RD, Relational Dynamics; CS, Communication Skills; IEPD, Internal and External Performance Design; SP, Stage Presence.

Parameter estimates in Table 6 revealed significant adjusted group differences across four CPP dimensions. The largest difference was observed in CS (*B* = −1.207, *t* = −6.445, *p* < 0.001, *η*^2^ = 0.379), followed by IEPD (*B* = −1.165, *t* = −5.197, *p* < 0.001, *η*^2^ = 0.284), CA (*B* = −0.658, *t* = −5.555, *p* < 0.001, *η*^2^ = 0.312), and RD (*B* = −0.561, *t* = −6.504, *p* < 0.001, *η*^2^ = 0.384). In contrast, SP showed no significant difference between groups (*B* = 0.090, *t* = 0.426, *p* = 0.672, *η*^2^ = 0.003).

**Table 6:** Adjusted parameter estimates of group effects on CPP dimensions.

Items	Parameter	*B*	*t*	Sig.	Partial eta squared
CA	[Group = Control]	−0.658	−5.555	<0.001	0.312
	[Group = Experimental]	0			
RD	[Group = Control]	−0.561	−6.504	<0.001	0.384
	[Group = Experimental]	0			
CS	[Group = Control]	−1.207	−6.445	<0.001	0.379
	[Group = Experimental]	0			
IEPD	[Group = Control]	−1.165	−5.197	<0.001	0.284
	[Group = Experimental]	0			
SP	[Group = Control]	0.09	0.426	0.672	0.003
	[Group = Experimental]	0			
Total Score	[Group = Control]	−3.672	−5.558	<0.001	0.312
	[Group = Experimental]	0			

CA, Character Analysis; RD, Relational Dynamics; CS, Communication Skills; IEPD, Internal and External Performance Design; SP, Stage Presence.

#### 3.2.2 Hypotheses testing

For H1 (CA), the analysis showed a significant group effect favouring the experimental cohort, *B* = −0.658, *t* = −5.555, *p* < 0.001, partial *η*^2^ = 0.312, indicating enhanced analytical engagement with character objectives and psychological detail. H2 (RD) was also supported, *B* = −0.561, *t* = −6.504, *p* < 0.001, partial *η*^2^ = 0.384, reflecting notable gains in sustaining authentic onstage relationships. For H3 (CS), the experimental group again outperformed the control group, *B* = −1.207, *t* = −6.445, *p* < 0.001, partial *η*^2^ = 0.379, showing improved articulation, projection, and communicative responsiveness. H4 (IEPD) yielded a significant advantage, *B* = −1.165, *t* = −5.197, *p* < 0.001, partial *η*^2^ = 0.284, indicating better integration of psychological intention and physical execution. H5 (SP) was not supported, *B* = 0.090, *t* = 0.426, *p* = 0.672, partial *η*^2^ = 0.003, with improvements comparable between groups, suggesting this dimension may be shaped by factors beyond the intervention. Overall, four of the five hypotheses were supported, confirming the intervention’s effectiveness in enhancing CA, RD, CS, and IEPD. The absence of a significant difference in SP points to the need for complementary pedagogical strategies to address this dimension more effectively in future training.

### 3.3 Qualitative analysis

#### 3.3.1 Overview of GTT’S contribution

**Figure 7:**
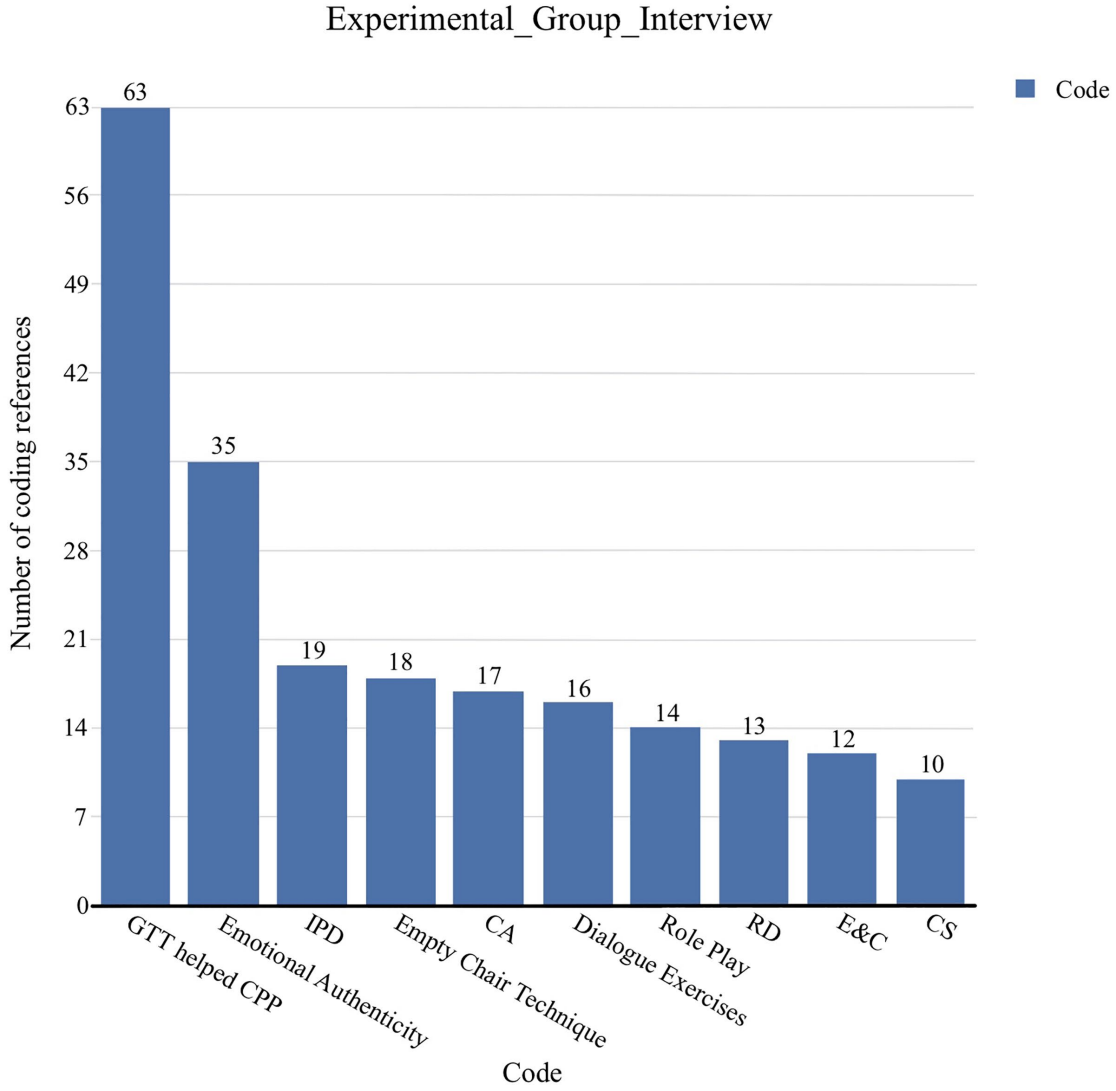
Experimental group interview themes codes.

**Figure 8:**
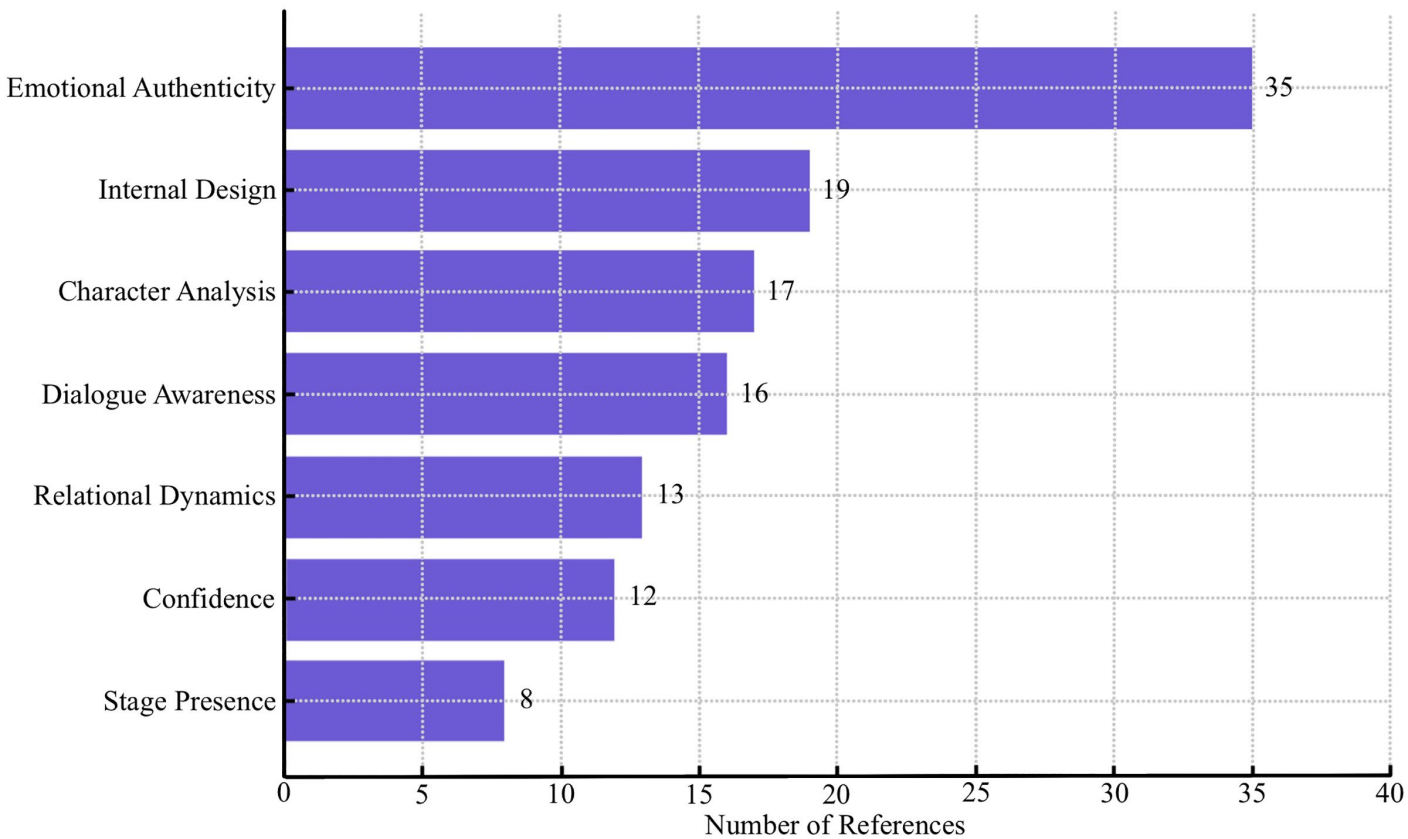
Coding frequency of GTT impact themes in student interviews.

NVivo analysis revealed strong consensus among both students and evaluators regarding GTT’s positive impact on CPP. The word cloud (Supplementary Figures) highlights terms such as *character*, *techniques*, *helped*, and *psychological*, indicating perceived enhancement of both psychological and expressive dimensions of acting. Thematic coding showed recurring patterns in how GTT influenced performance development. Beyond the overall category *GTT helped CPP* (63), the most frequent references were *Emotional Authenticity* (35), *Internal Design* (19), and *CA* (17). *Dialogue Exercises* (16) and *Role Play* (14) were often cited as effective for exploring character depth and fostering responsiveness (Figure 7). SP was not among the top-ranked codes (Figure 8), aligning with quantitative results showing no significant SP effect. The prominence of emotional authenticity and motivation aligned with gains observed in CA and IEPD, reinforcing the link between qualitative themes and quantitative outcomes. Students most frequently associated GTT with enhancing emotional authenticity, internal performance design, and character analysis—concrete experiences repeatedly identified during discussion. Experts corroborated these perceptions, noting observable differences in emotional truth, psychological grounding, and relational dynamics between groups. For example, students highlighted “how GTT contributes to authenticity in scenes,” whilst experts “assessed GTT’s influence across the five CPP dimensions.”

“It made the character feel more real and helped me stay connected to them.” (Student 6).

“I started building the scenes from the character’s motivations, and that changed how I acted in each moment.” (Student 3).

“It was not only memorizing lines but also experiencing what the character felt inside.” (Student 5).

“I noticed that the students in the experimental group tended to take more emotional risks. Their work felt less mechanical, more lived.” (Expert 3).

“They became less focused on external imitation and more on building from inner motivation.” (Expert 2).

“Based on student evaluations, it’s clear that Gestalt techniques are helpful for certain fundamental aspects of role development.” (Expert 1).

Overall, both data sets indicate that GTT enriched emotional authenticity (combined CA and IEPD) of CPP whilst functioning as a complementary approach to Stanislavski-based training. These qualitative findings align closely with the quantitative results reported earlier, reinforcing the integrative pedagogical potential of GTT in undergraduate acting education. These outcomes prepare the ground for a more detailed analysis of student perspectives.

#### 3.3.2 Student perspectives on GTT’S impact

Analysis of the experimental group interview data revealed consistent and multidimensional recognition of the value GTT brought to performance development. The *Empty Chair Technique* accounted for the largest share, with 18 codes, followed by *Dialogue Exercises* (16) and *Role Play* (14), whilst *Exaggeration* ranked lowest (see Supplementary Figures). A few students also noted limitations of this technique. The coding of specific GTT impact themes showed that *Emotional Authenticity* was referenced most frequently (35), followed by *Internal Design* (19), *CA* (17), and *Dialogue Awareness* (16). Less frequent but still noteworthy were *Confidence* (12) and *SP* (8). A detailed breakdown of coding frequency is presented in Figure 8. Students repeatedly described GTT as enabling them to move from surface-level representation to deeper experiential engagement with their roles. *Emotional authenticity* emerged as the dominant theme:

“GTT supported our process and helped intensify that sense of authenticity during rehearsal and performance.” (Student 7).

“That really helped us dig into the inner layers of the character.” (Student 5).

In terms of *Internal Design* and *Character Analysis*, students highlighted a greater awareness of their characters’ psychological landscapes:

“It made me think about the character’s inner world, not just the lines. Like, what do they want? What are they afraid of?” (Student 1).

“I started building the scenes from the character’s motivations, and that changed how I acted in each moment.” (Student 3).

For *Dialogue Awareness* and relational dynamics, students reported more attentive listening and responsiveness in scene work:

“I used to just wait for my turn to talk, but now I notice what the other character is really giving me.” (Student 4).

“It helped me find a more natural flow when talking to other characters—and even when just speaking as myself.” (Student 2).

Although Confidence and Stage Presence appeared less frequently in the coding, they were still perceived as meaningful outcomes:

“That gave us confidence when performing.” (Student 1).

“It helped us dare to express ourselves more boldly on stage, which in turn improved our control and presence.” (Student 4).

Overall, these perspectives suggest that GTT supported growth across affective, cognitive, and behavioural dimensions of CPP. The emphasis on emotional truth, coherent internal logic, and responsive interaction mirrors the improvements identified in the quantitative analysis, underscoring the complementary value of GTT in undergraduate acting training.

#### 3.3.3 Expert insights on GTT integration and observations

**Figure 9:**
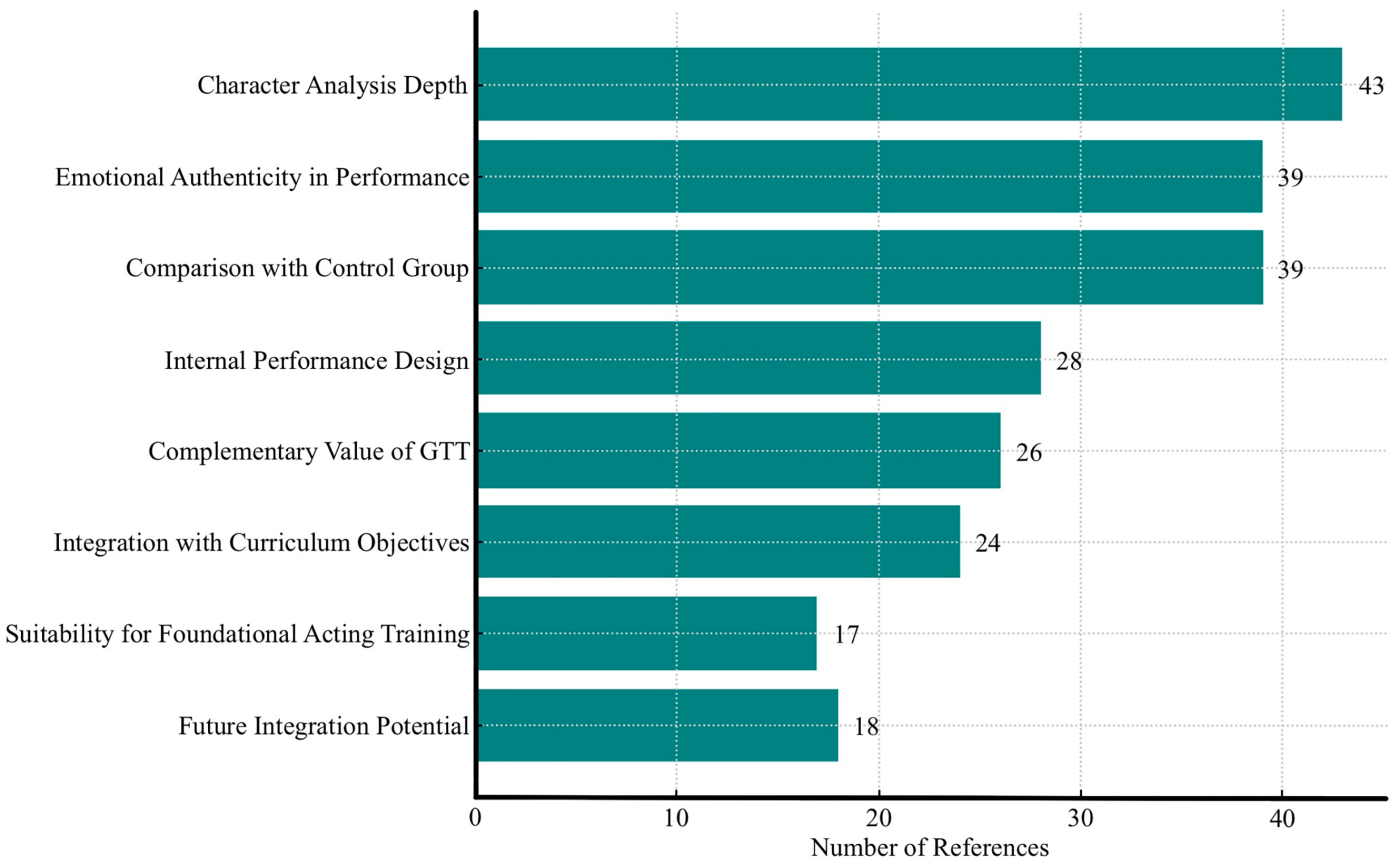
Expanded expert coding frequencies on GTT.

The expert interviews provided multi-dimensional perspectives on the pedagogical value and observable effects of GTT in undergraduate acting training. As shown in Figure 9, NVivo coding indicated that *Character Analysis Depth* (43 references) and *Emotional Authenticity in Performance* (39) were the most frequently noted strengths of the experimental group. *Internal Performance Design* (28) and *Comparison with the Control Group* (39) were also highly coded, whilst *Complementary Value of GTT* (26) reflected a consensus that GTT should be integrated alongside, rather than in place of, Stanislavski-based methods.

Experts observed that GTT fostered richer psychological engagement and more coherent scene work. For instance:

“When students use the empty chair to dialogue with a past version of their character, it promotes deeper self-reflection and analysis.” (Expert 2).

“Students from the experimental group demonstrated more layered backstories, and their actions on stage aligned better with psychological motivation than those in the control group.” (Expert 2).

“Many students in the experimental group seemed genuinely moved during their scenes, which shows emotional authenticity.” (Expert 1).

Whilst the impact on *External Performance Design* (9 references) and *SP* (3) was acknowledged, experts regarded these effects as more indirect. This may help explain why the quantitative results showed no significant effect for SP. As Expert 1 cautioned:

“Even if the emotional content is more sincere, the impact of the scene still depends heavily on blocking, voice, timing—and crucially, on the director’s shaping.”

Pedagogical recommendations emerging from these insights are summarised. The overall hierarchy sunburst coding (see Supplementary Figures) shows that experts strongly affirmed the impact of GTT on CPP, with a large proportion of references addressing observed effects on students. At the same time, experts highlighted key pedagogical challenges and offered targeted recommendations. Core suggestions included embedding GTT systematically into foundational acting courses, providing specialized teacher training, and gradually extending its use to voice, movement, and devised theatre components. Their reflections were closely tied to the interview focus areas “Identifying practical limitations, implementation difficulties, or technical concerns in applying GTT” and “Exploring the sustainability of GTT in actor training and future directions for pedagogy and research,” where experts articulated both the challenges and the potential pathways for long-term integration. For example:

“Gestalt techniques should be integrated at the entry level of actor training so that habits of authenticity are established from the start.” (Expert 1).

“The application of GTT in foundational actor training can provide substantial benefits. It contributes to more rigorous character analysis, facilitates a deeper understanding of relational dynamics, and supports the exploration of character backstories.” (Expert 1).

“These techniques are integrated with traditional acting pedagogy in a way that fosters mutual reinforcement and coherence.” (Expert 2).

“It’s important to explore how psychological techniques can be adapted to suit different classroom environments and student needs.” (Expert 2).

“These methods are not only relevant to acting scenes—they should also extend to voice and movement classes, where presence and awareness are equally critical.” (Expert 3).

“Gestalt methods focus heavily on internal processes—perhaps they can also improve external expression and packaging of the character.” (Expert 3).

“The challenge for instructors is significant—they must first become familiar with these theories and techniques. Students also need to acknowledge that psychological understanding is crucial for acting.” (Expert 3).

## 4 Discussion

### 4.1 Interpretation of key findings

This study set out to evaluate whether integrating GTT with traditional Stanislavsky-based training produces measurable improvements in CPP and to explore participants’ perceptions of such integration. The quantitative results directly address RQ1 and the first research objective, showing that the experimental group achieved significantly higher CPP post-test scores. In particular, the dimensions of CA, RD, CS, and IEPD were significantly enhanced compared with the control group. These improvements align with previous research that links experiential, present-centred methods to deeper emotional connection and psychological coherence in performance (Fels, 2019; Habsy et al., 2024; Austin and Austin, 2022; Josek et al., 2023; Mann, 2020). The absence of significant difference in SP echoes prior findings that stage charisma is more strongly influenced by sustained technical work in voice, movement, and spatial command (Peppler et al., 2023; Streitberger, 2017). This suggests that GTT may primarily strengthens internal and foundational dimensions rather than directly influencing stage dominance. This study’s findings are grounded in theatrical traditions that prioritize psychological realism and emotional authenticity (e.g., Chekhov, Strindberg). Future research should explore whether these benefits extend to other performance genres.

The qualitative analysis further illuminates these statistical patterns and answers RQ2 and the second research objective. Students reported that GTT shifted their approach from “delivering lines” to “inhabiting the character’s inner life,” with several noting how techniques such as the Empty Chair and Dialogue Exercises helped uncover motives and relational intentions. These reflections are consistent with the view of Garrett (2024) and Alcott (2024), who argue authentic stage communication arises from an actor’s capacity to respond truthfully to imagined circumstances. Expert insights indicate that GTT are most effective when introduced early in actor training, as this timing helps establish habits of authenticity from the outset. When integrated with Stanislavski-based methods, GTT enhances character analysis, deepens relational understanding, and supports backstory exploration, whilst also extending its value to voice and movement classes where presence and awareness are essential. Experts further emphasized that GTT links internal processes with external expression, thereby promoting more holistic actor development. At the same time, they cautioned that its successful application requires instructors to develop sufficient familiarity with the methods and adapt them to varied classroom contexts and student needs (Berry and Brown, 2019). Experts corroborated these perceptions, observing that experimental group performances displayed more coherent psychological through-lines and more nuanced interaction, which argument that believable character work depends on the integration of emotional truth and clear motivation (Gaurav and Bhardwaj, 2024; Madoyan, 2023; McFarren, 2008).

It produced measurable improvements in core CPP dimensions, most notably in emotional authenticity (CA, IEPD) and relational engagement (RD, CS). Both students and experts recognised these gains, highlighting the effectiveness of GTT as a complement to Stanislavsky-based training.

### 4.2 Integration with existing literature and pedagogical implications

The present findings extend prior scholarship on actor training by evidencing that GTT can address well-documented limitations of traditional Stanislavsky-based pedagogy. However, Stanislavsky’s system offers a structured pathway to building a role, it can fall short in facilitating sustained emotional accessibility and responsive interaction, particularly among novice actors (Dobreva-Hristova, 2022; Zhigang, 2022). The significant improvements observed in CA, RD, IPED and CS in the experimental group suggest that GTT’s present-centred, experiential focus (Atkins, 2022; Hart, 2020; Stanislavskij, 2021) complements the Stanislavsky framework by anchoring technical preparation in genuine emotional experience.

These results also resonate with Berry and Brown’s (2019) assertion that truthful stage communication depends on an actor’s capacity to connect inner impulses with outward expression. Techniques such as the Empty Chair and Dialogue Exercises directly engaged students in exploring unresolved conflicts and relational objectives, which experts identified as leading to more coherent psychological through-lines in performance (Ndhlovu and Mwanza, 2024; Tucker, 2023). Similarly, the Exaggeration Technique and Role Play have enhanced IEPD which aligns the view that deliberate physical amplification can strengthen the integration of inner emotional states with physical embodiment (Case, 2018; Pugh et al., 2001; Nair, 2019).

From a pedagogical standpoint, the experts highlighted the need to integrate GTT systematically into actor training. They emphasised introducing it early in foundational courses to cultivate habits of emotional truth, supporting instructors through specialised training to ensure competent delivery, and extending its application across movement, voice, and devised theatre. This staged integration reflects Goodall’s (2008) and Mirodan’s (2015) emphasis on developing stage presence and performance coherence through sustained, multi-faceted training.

In sum, this study positions GTT not as a replacement for traditional acting systems but as a complementary methodology that strengthens psychological grounding and relational responsiveness. By bridging internal authenticity with technical craft, GTT integration offers a viable pedagogical model for cultivating well-rounded, emotionally present actors within undergraduate programmes.

### 4.3 Limitations

The interpretation of these findings should take into account several limitations. First, whilst the intervention was delivered within class hours, students’ opportunities for additional rehearsal outside the classroom were uneven. Differences in access to rehearsal spaces, availability of peers, and the broader learning environment may have shaped how effectively skills were reinforced beyond the formal sessions, potentially influencing outcomes across groups. Second, the results are also conditioned by individual variation in learning capacity and receptiveness to new methods. Some students were able to adapt quickly to the Gestalt techniques, whilst others required more time and support, leading to variability in the extent to which training gains were consolidated. A further limitation relates to the potential influence of social desirability bias. Although acting students are generally more open in self-expression, the presence of the instructor during interviews may have encouraged participants to emphasise positive experiences, thereby shaping the qualitative responses. Taken together, these factors highlight that both environmental conditions and individual readiness, as well as context-dependent response tendencies, can significantly affect the impact of pedagogical interventions. They also underscore the need for future studies to account for extracurricular rehearsal opportunities, baseline differences in student ability, and potential interview bias when evaluating the integration of GTT into actor training.

### 4.4 Directions for future research

Future research could advance this study in several important directions. One promising direction is to enrich the evaluation of CPP by incorporating structured audience feedback, thereby offering a multi-perspective assessment of performance outcomes and better capturing the communicative dimension of stage work. In addition, systematic classroom observation records would complement interview data, and when analysed alongside student and expert perspectives, such observations would enable methodological triangulation (Denzin, 2012), enhancing the validity of qualitative findings and providing a more comprehensive account of how GTT functions in practice. Another important avenue concerns longitudinal research designs, which could test whether gains in emotional authenticity and relational engagement are sustained over time and become embedded in students’ habitual performance practice. These designs would also help to determine whether stage presence can be strengthened through extended training. Prior work in educational psychology suggests that embodied and relational competencies often require prolonged, distributed practice to stabilise (Rowat et al., 2021). Finally, broadening the scope of Gestalt methods under investigation may yield further insights. Beyond the Empty Chair, Dialogue Exercises, Role Play, and Exaggeration, techniques such as Reversal (Apter, 2001) and Guided Fantasy (Erreich, 2015) may reveal additional pathways for strengthening dimensions of CPP. Exploring how these methods interact with voice, movement, and devised theatre training could further illuminate the pedagogical potential of GTT.

## 5 Conclusion

This study examined the integration of GTT with Stanislavski-based training to enhance CPP. Drawing on a mixed-methods design, the findings confirmed significant gains in key CPP dimensions CA, RD, CS, and IEPD. Both students and experienced evaluators emphasised that these improvements were closely linked to heightened emotional authenticity (CA, IEPD) and relational engagement (RD, CS) At the same time, SP did not show a significant effect, underscoring that this dimension may require longer-term development through sustained work in voice, movement, and spatial command. Qualitative insights further highlighted that GTT complements but does not replace the technical rigour of traditional actor training. Instead, it provides a psychologically grounded foundation that enriches the Stanislavski system, addressing gaps in cultivating authentic responses and relational depth.

Taken together, these results demonstrate that GTT integration not only advances performance skills but also broadens the pedagogical repertoire for actor training. By embedding GTT into foundational curricula, equipping instructors with targeted training, and extending applications to voice, movement, and devised theatre, educators can develop more responsive and psychologically attuned performers. Future research should test these findings across broader contexts and longer timescales, but the evidence presented here establishes GTT as a robust and transferable framework for strengthening the foundations of CPP.

## Data Availability

The datasets presented in this study can be found in online repositories. The names of the repository/repositories and accession number(s) can be found in the article/Supplementary material.
