# Supplementary material for: Integrating gestalt therapeutic techniques enhances character portrayal in undergraduate acting students
Source: Front Psychol. 2026 Mar 27;17:1697077. doi: 10.3389/fpsyg.2026.1697077 (PMC13067942; doi:10.3389/fpsyg.2026.1697077)
Supplement: Supplementary file 1 [file Supplementary_File_1.pdf]

Supplementary Material

1 Supplementary Data

1.1 Power analysis results of Sample Size (G*Power 3.1)

Analysis type	Parameters	Result	Interpretation
**A priori**	f = 0.35; α = .05; power = .80; 2 groups; 1 covariate	Required N = **67**	Actual N = **71** > 67
**Sensitivity**	N = **71**; α = .05; power = .80	Detectable effect **f ≥ 0.337** ($\eta^2 \approx .10$)	Detects medium effects and above
**Post hoc**	f = 0.35; N = **71**; α = .05; 2 groups; 1 covariate	Achieved power = **0.828**	Above .80 threshold

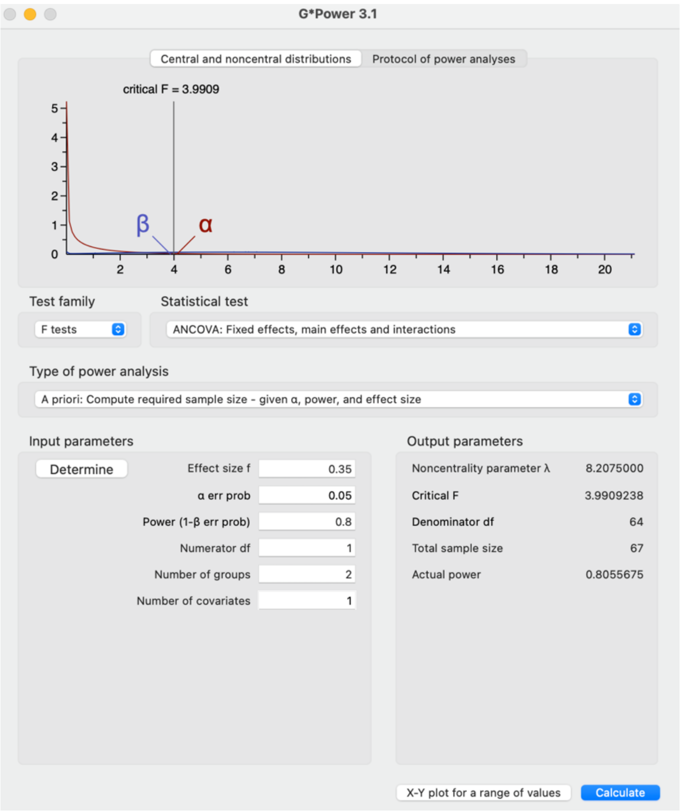

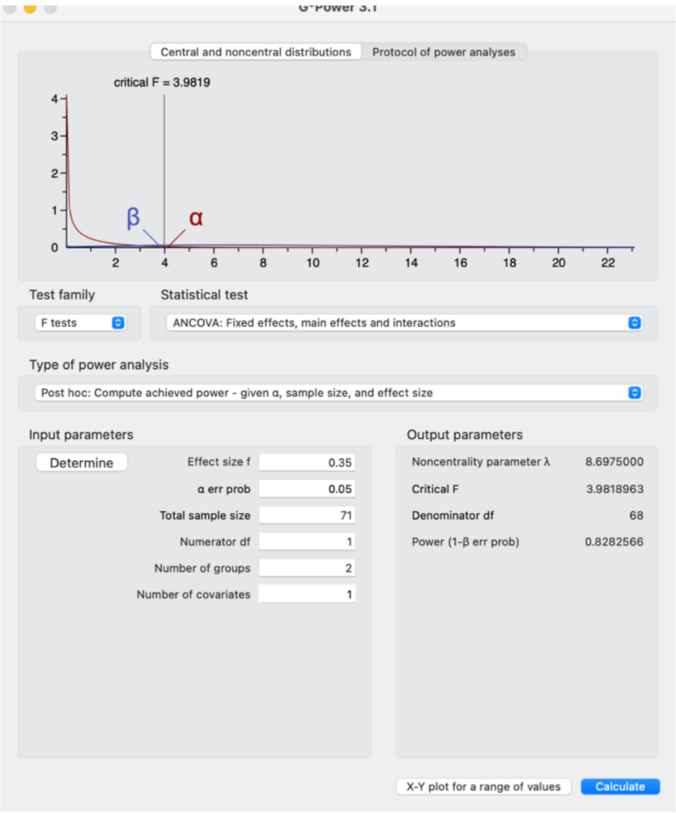

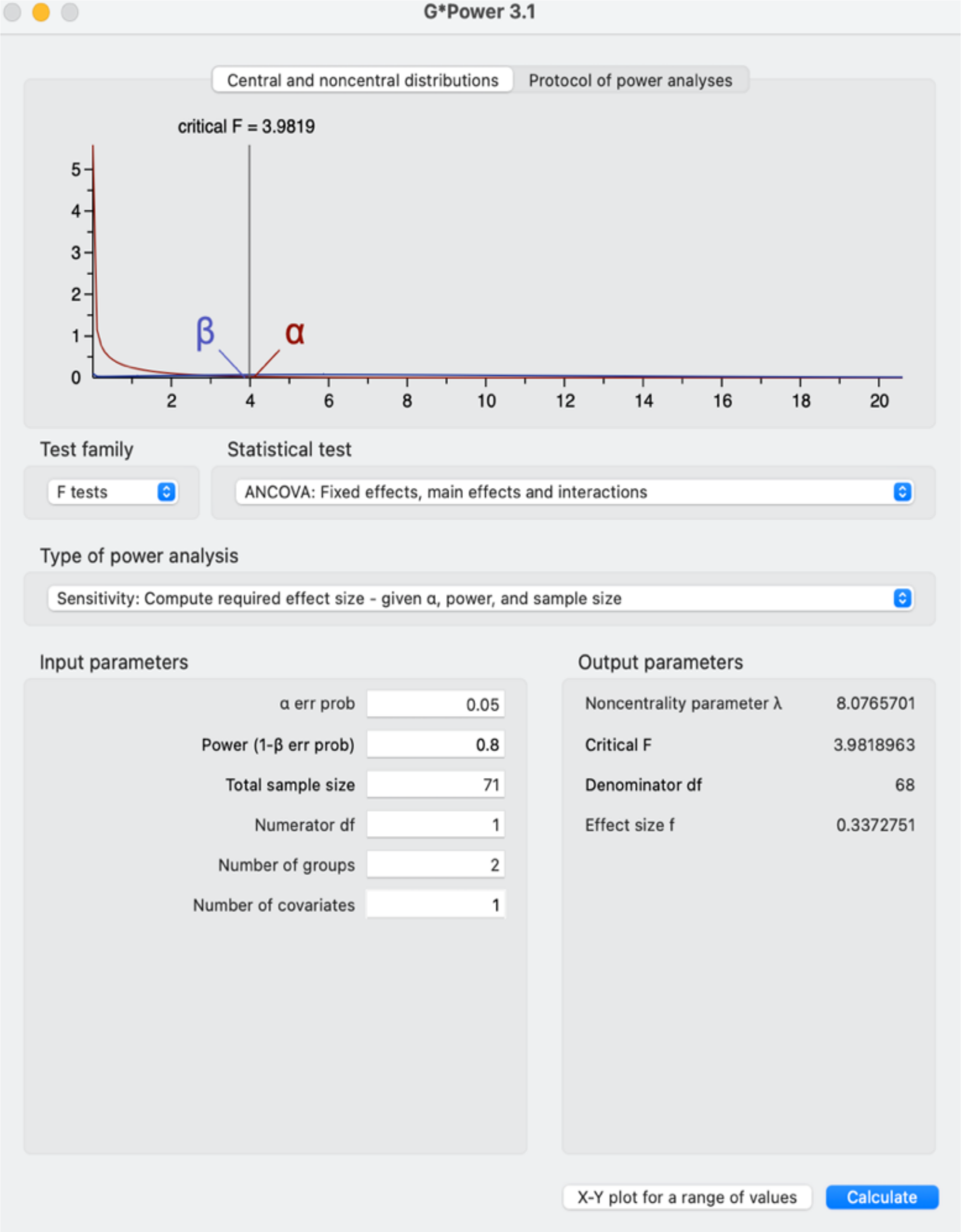
G*Power 3.1
Central and noncentral distributions
Protocol of power analyses
critical F = 3.9819
β
α
Test family
F tests
Statistical test
ANCOVA: Fixed effects, main effects and interactions
Type of power analysis
Sensitivity: Compute required effect size - given α, power, and sample size
Input parameters
α err prob 0.05
Power (1-β err prob) 0.8
Total sample size 71
Numerator df 1
Number of groups 2
Number of covariates 1
Output parameters
Noncentrality parameter λ 8.0765701
Critical F 3.9818963
Denominator df 68
Effect size f 0.3372751
X-Y plot for a range of values
Calculate

1.2 Evaluation of the questionnaire validation

FOR EXPERT PANEL

01

Item-Objective Congruence (IOC) Rating Table for Expert Semi-Structured Interview

Explanation:

This table evaluates the relevance of the interview questions to the study's objectives using the Item-Objective Congruence (IOC) method. Each item is rated by three experts on a scale of -1 to 1:

- **-1**: The item is not relevant to the objective.
- **0**: The item is somewhat relevant to the objective.
- **1**: The item is highly relevant to the objective.

IOC Results: Expert Semi-Structured Interviews

Item	**Expert 1 (-1, 0, 1)**	**Expert 2 (-1, 0, 1)**	**Expert 3 (-1, 0, 1)**	Number in Agreement	Item IOC
1	1	1	1	3	1.00
2	1	1	0	2	0.67
3	1	1	1	3	1.00
4	1	1	1	3	1.00
5	1	1	1	3	1.00
6	1	1	1	3	1.00
7	1	1	1	3	1.00
8	1	1	1	3	1.00
9	1	1	1	3	1.00
10	1	1	1	3	1.00

Formula for Item IOC: $$\text{Item IOC} = \frac{\text{Sum of Expert Ratings}}{\text{Number of Experts}}$$

02

Item-Objective Congruence (IOC) Rating Table for Focus Group Interview Questions

Explanation:

This table evaluates the relevance of the interview questions to the study's objectives using the Item-Objective Congruence (IOC) method. Each item is rated by three experts on a scale of -1 to 1:

- **-1**: The item is not relevant to the objective.
- **0**: The item is somewhat relevant to the objective.
- **1**: The item is highly relevant to the objective.

The average score across the three experts determines the final validity score of each item.

IOC Results: Focus Group Interviews

Item	**Expert 1 (-1, 0, 1)**	**Expert 2 (-1, 0, 1)**	**Expert 3 (-1, 0, 1)**	Number in Agreement	Item IOC
1	1	1	1	3	1.00
2	1	1	1	3	1.00
3	1	1	0	2	0.67
4	1	1	1	3	1.00
5	1	1	1	3	1.00
6	1	1	1	3	1.00
7	-1	0	-1	1	-0.67
8	1	1	1	3	1.00
9	1	1	1	3	1.00
10	0	1	1	2	0.67
11	1	1	1	3	1.00

Formula for Item IOC: $$\text{Item IOC} = \frac{\text{Sum of Expert Ratings}}{\text{Number of Experts}}$$

Note. Item 7 did not meet the acceptable IOC threshold (IOC = –0.67) and was therefore removed from the instrument.

1.3 ANCOVA Results

1) Descriptive Statistics of Control Group Pre-test

	N	Minimum	Maximum	Mean	Std. Deviation
Character Analysis （15%）	35	8	13	10.24	0.911
Relational Dynamics（15%）	35	9	13	10.93	0.895
Communication Skills（25%）	35	16	21	17.69	1.176
Internal and External Performance Design（25%）	35	16	21	17.04	1.093
Stage Presence and Engagement （20%）	35	11	16	13.32	1.052
Total_Score（100 points）	35	62	83	69.23	4.473

2) Descriptive Statistics of Control Group post-test

	N	Minimum	Maximum	Mean	Std. Deviation
Character Analysis （15%）	35	10	13	11.38	0.761
Relational Dynamics（15%）	35	10	13	11.54	0.582
Communication Skills（25%）	35	16	22	19.49	1.135
Internal and External Performance Design（25%）	35	16	22	19.15	1.084
Stage Presence and Engagement （20%）	35	12	18	14.98	1.177
Total_Score（100 points）	35	64	88	76.53	4.297

3) Descriptive Statistics of Experimental group pre-test

Character Analysis （15%）	36	9	13	10.55	1.016
Relational Dynamics（15%）	36	8	13	10.57	0.954
Communication Skills（25%）	36	14	21	16.57	1.649

Internal and External Performance Design（25%）	36	14	20	16.1	1.531
Stage Presence and Engagement （20%）	36	10	15	13.18	1.078
Total_Score（100 points）	36	57	81	66.97	4.856

4) Descriptive Statistics of Experimental group pre-test

	N	Minimum	Maximum	Mean	Std. Deviation
Character Analysis （15%）	36	11	14	12.19	0.593
Relational Dynamics（15%）	36	10	13	11.93	0.538
Communication Skills（25%）	36	16	23	19.92	1.32
Internal and External Performance Design（25%）	36	15	23	19.76	1.259
Stage Presence and Engagement （20%）	36	12	18	14.78	1.166
Total_Score（100 points）	36	66	90	78.58	4.284

5) Tests of Homogeneity of Regression Slopes for CPP Dimensions

**Source**	**Type III Sum of Squares**	**Mean Square**	**F**	**P**	**Partial Eta Squared**
Group * Character Analysis（PRE）	0.399	0.399	1.663	0.202	0.024
Group * Relational Dynamics（PRE）	0.333	0.333	2.683	0.106	0.038
Group * Communication Skills（PRE）	0.545	0.545	1.013	0.318	0.015
Group * Internal and External Performance Design（PRE）	0.944	0.944	1.197	0.278	0.018
Group * Stage Presence （PRE）	0.111	0.111	0.14	0.71	0.002
Group * Total Score（PRE）	2.379	2.379	0.322	0.572	0.005

6) **ANCOVA Results for Post-Test Scores with Pre-Test Scores as Covariates**

	Source	Type III Sum of Squares	F	Sig.	Partial Eta Squared
Character Analysis	Character Analysis（PRE）	15.521	64.023	<.001	0.485
	Group	7.48	30.856	<.001	0.312
Relational Dynamics	Relational Dynamics（PRE）	12.997	102.296	<.001	0.601
	Group	5.375	42.303	<.001	0.384
Communication Skills	Communication Skills（PRE）	68.196	126.682	<.001	0.651
	Group	22.359	41.535	<.001	0.379
Internal and External Performance Design	Internal and External Performance Design（PRE）	41.703	52.759	<.001	0.437
	Group	21.346	27.006	<.001	0.284
Stage Presence	Stage Presence （PRE）	41.412	52.88	<.001	0.437
	Group	0.142	0.181	0.672	0.003
Total Score	Total Score（PRE）	773.39	105.852	<.001	0.609
	Group	225.73	30.895	<.001	0.312

7) Adjusted Parameter Estimates of Group Effects on CPP Dimensions

	Parameter	B	t	Sig.	Partial Eta Squared
Character Analysis	[Group=Control]	-0.658	-5.555	<0.001	0.312
	[Group=Experimental]	0			
Relational Dynamics	[Group=Control]	-0.561	-6.504	<0.001	0.384
	[Group=Experimental]	0			
Communication Skills	[Group=Control]	-1.207	-6.445	<0.001	0.379
	[Group=Experimental]	0			
Internal and External Performance Design	[Group=Control]	-1.165	-5.197	<0.001	0.284
	[Group=Experimental]	0			
Stage Presence	[Group=Control]	0.09	0.426	0.672	0.003
	[Group=Experimental]	0			
Total Score	[Group=Control]	-3.672	-5.558	<0.001	0.312
	[Group=Experimental]	0			

2 Translation

2.1 Translation of CPP Assessment Documents (SUMC Approval)

Sichuan University of Media and Communications Examination

Character Portrayal Proficiency Assessment Form

Academic Year: 2024–2025 | **Semester:** 2
Course Code: 93142405
Course Name: One-Act Play Performance
Examination Grade Level: 2023 Cohort
Course Category:

表演专业

- General Required ☐
- General Elective ☐
- Basic Major ☐
- Core Major ☑
- Major Elective ☐
- Other: ______

Review Content | Evaluation Levels

Criteria	Evaluation
**1. Alignment of assessment content with curriculum outline**	Aligned ☑
**2. Scientific and rational examination method**	Scientific & Rational ☑
**3. Operability of assessment process**	Highly Operable ☑
**4. Overall difficulty level of the assessment requirements**	Moderate ☑

Review Comments

Program Director (Signature): Qi Yan
Date: December 6, 2024

Approval Comments

Dean of the College (Signature): Renhua Na
Date: December 6, 2024

☑ Approved Assessment Scheme
☑ Approved Assessment Scheme

I, Ji Xiangdi, translator of E-TRANSTAR (Beijing) Information Technology Co., Ltd., confirm this is a true and accurate translation of the original document.

CATTI Certificate No.: 201906009370001040 Organization: E-TRANSTAR (Beijing) Information Technology Co., Ltd.

Tel: 010-60537024 Email: 3004146455@qq.com

Organization Address: 201-212057, Zone 6, Pinggu Park, Zhongguancun Science and Technology Park, Pinggu District, Beijing

Signature: Ji Xiangdi Date of Translation: January 22, 2025

E-Transtar (Beijing) Information Technology Co., Ltd. 易思达（北京）信息技术有限公司 翻译专用章 Valid for Translation Only

Examination Scheme for Practical (Performance-Based) Courses

Course Code: 93142405

Course Name: One-Act Play Performance

表演专业

Grade Level: 2023 Cohort

Major: Acting

I. Examination Topic

"The Last Empress and Der Ling" – Character Portrayal

II. Examination Content and Methods

Content:

1. Character Analysis
2. Depiction of Character Relationships
3. Communication Skills
4. Internal and External Performance Design
5. Stage Presentation

Method:
The examination will be conducted through a live performance of the one-act play.

III. Examination Date

June 12, 2025

IV. Examination Results and Presentation

Students will complete their one-act play performances live on stage. Each student's performance will be evaluated and scored on-site by instructors. The performances will also be recorded and archived for reference.

I, Ji Xiangdi, translator of E-TRANSTAR (Beijing) Information Technology Co., Ltd., confirm this is a true and accurate translation of the original document.

CATTI Certificate No.: 201906009370001040 Organization: E-TRANSTAR (Beijing) Information Technology Co., Ltd.

Tel: 010-60537024 Email: 3004146455@qq.com

Organization Address: 201-212057, Zone 6, Pinggu Park, Zhongguancun Science and Technology Park, Pinggu District, Beijing

Signature: Ji Xiangdi Date of Translation: January 22, 2025

E-Transtar (Beijing) Information Technology Co., Ltd. 易思达（北京）信息技术有限公司 翻译专用章 Valid for Translation Only

Sichuan University of Media and Communications

Character Portrayal Proficiency Assessment Criteria

表演专业

Course Name: One-Act Play Performance

Grade Level: 2023 Cohort

Assessment Topic, Format, and Method Description:

1. Assessment Format: Character Analysis written report and live one-act play performance.

2. Student live performance:

Students will present assigned characters in the one-act play.

Instructors will evaluate students' mastery of course content based on their live performance.

Assessment Criteria and Weighting Total Score: 100 points

Criteria	Weight	Scoring Guidelines
**1. Character Analysis**	**15%**	**11-15 points**: Provides an in-depth understanding of the character's motivations, background, and context.<br>**6-10 points**: Demonstrates partial understanding with general insights.<br>**0-5 points**: Displays limited understanding, lacks depth or clarity.
**2. Depiction of Character Relationships**	**15%**	**11-15 points**: Accurately and convincingly portrays relationships with other characters.<br>**6-10 points**: Shows general ability to depict relationships but lacks depth or consistency.<br>**0-5 points**: Portrayal of relationships is unclear or lacks authenticity.
**3. Communication Skills**	**25%**	**20-25 points**: Demonstrates highly effective verbal and non-verbal communication, including clear articulation, appropriate gestures, and expressive body language.<br>**10-19 points**: Communication is moderately effective, with some inconsistencies in delivery or expression.<br>**0-9 points**: Ineffective communication, lacking clarity, expressiveness, or appropriate non-verbal cues.
**4. Internal and External Performance Design**	**25%**	**20-25 points**: Successfully integrates emotional depth with physical movement, creating a cohesive and engaging performance.<br>**10-19 points**: Shows partial integration of emotional and physical performance, with room for improvement.<br>**0-9 points**: Limited connection between emotional expression and physical movement, leading to a disjointed performance.
**5. Stage Presence**	**20%**	**15-20 points**: Presents a complete and polished performance with clear rhythm and strong audience impact.<br>**8-14 points**: Presents a mostly complete performance, but rhythm and engagement are inconsistent.<br>**0-7 points**: Performance lacks completion, rhythm, and audience engagement.

Date: December 6, 2024

I, Ji Xiangdi, translator of E-TRANSTAR (Beijing) Information Technology Co., Ltd., confirm this is a true and accurate translation of the original document.

CATTI Certificate No.: 201906009370001040 Organization: E-TRANSTAR (Beijing) Information Technology Co., Ltd.

Tel: 010-60537024 Email: 3004146455@qq.com

Organization Address: 201-212057, Zone 6, Pinggu Park, Zhongguancun Science and Technology Park, Pinggu District, Beijing

Signature: Ji Xiangdi Date of Translation: January 22, 2025

E-Transtar (Beijing) Information Technology Co., Ltd. 易传思达（北京）信息技术有限公司 翻译专用章 Valid for Translation Only

Sichuan University of Media and Communications

Character Portrayal Proficiency Evaluation Form

表演专业

**Student ID**	
**Name**	
**Course Name**	One-Act Play Performance
**Class**	2023 Cohort Acting Major

Assessment Topic, Format, and Method Description:

1. Assessment Format: Written character analysis report and one-act play performance.

2. Student Live Performance: Students present their assigned roles in a live performance.

3. Evaluation Method: Instructors assess students' mastery of course content based on their performance.

No.	Assessment Criteria	Weight	Score
1	Character Analysis	15%	
2	Depiction of Character Relationships	15%	
3	Communication Skills	25%	
4	Internal and External Performance Design	25%	
5	Stage Presentation	20%	
	**Total**	**100%**	

Notes:

Instructor Signature: ___________________________

I, Ji Xiangdi, translator of E-TRANSTAR (Beijing) Information Technology Co., Ltd., confirm this is a true and accurate translation of the original document.

CATTI Certificate No.: 201906009370001040 Organization: E-TRANSTAR (Beijing) Information Technology Co., Ltd.

Tel: 010-60537024 Email: 3004146455@qq.com

Organization Address: 201-212057, Zone 6, Pinggu Park, Zhongguancun Science and Technology Park, Pinggu District, Beijing

Signature: Ji Xiangdi Date of Translation: January 22, 2025

E-Transtar (Beijing) Information Technology Co., Ltd. 易思达（北京）信息技术有限公司 翻译专用章 Valid for Translation Only 1101090075601

2.2 Translation of Interviews Questions

探索格式塔治疗技术在本科表演学生角色塑造能力中的整合应用

专家半结构化访谈问题

(Expert Semi-Structured Interview Questions)

编号	问题（Questions）
1	根据您的评估，格式塔治疗技术（GTT）如何影响了实验组学生角色的塑造能力？
2	您如何评价GTT对学生理解角色和人物关系的促进作用？
3	您如何评价GTT 的对话练习？它对提升了学生语言和非语言交流能力的关键点是什么？
4	GTT的夸张技术和斯坦尼斯拉夫斯基的行动技巧有什么区别和优势？
5	GTT 中的角色扮演对帮助学生建立可信的角色互动是否比传统方法更好？
6	学生作品呈现中内外部表演设计情况实验组与控制组有哪些异同（优势或者不足）？
7	在学生呈现的情感强烈或高潮戏份中，实验组同学的舞台真实感是否更好？
8	从两组的舞台呈现来看，格式塔技术是否增强了学生的自信心，并提升了学生对舞台的掌控能力？
9	在课程中融合GTT应用到表演课堂要注意哪些事项？未来的挑战表现在哪些方面？
10	GTT对于表演教学否具有更广泛的应用价值？未来的研究应探索哪些方向？

本人，易传思达（北京）信息技术有限公司的翻译季相娣，确认这是对原始文件的真实准确的翻译。
CATTI 证书编号：201906009370001040
组织机构：易传思达（北京）信息技术有限公司
电话：010-60537024　邮箱：3004146455@qq.com
组织地址：北京市平谷区中关村科技园平谷园 6 区
签名：　翻译日期：2025 年 2 月 6 日

探索格式塔治疗技术在本科表演学生角色塑造能力中的整合应用

焦点小组访谈问题
（Focus Group Interview Questions）

编号	问题（Questions）
1	你是用什么方法分析《德龄与慈禧》中自己角色的人物的？
2	空椅子技术（Empty Chair Technique）如何帮助你理解角色的情感和决策过程？
3	哪些对话练习（Dialogue Exercises）最有效地提升了你的语言和非语言交流能力？
4	夸张技巧（Exaggeration Technique）如何帮助你优化肢体表现力？
5	角色扮演（Role Play）如何帮助你建立可信的角色互动？
6	格式塔技术（Gestalt Techniques）如何影响你在剧本中表现角色关系的？
7	你能否描述你在表演呈现，格式塔的方法对肢体语言外化内部情感有怎样的帮助？
8	格式塔这些技术如何影响你表演中的特定时刻，例如激烈的对抗场景或内心独白？
9	在情感强烈或高潮戏份中，哪些方法帮助您建立真实感？
10	相比较传统方法，格式塔技术是否增强了你的自信心，它如何提升你对舞台的掌控能力？

本人，易传思达（北京）信息技术有限公司的翻译季相娣，确认这是对原始文件的真实准确的翻译。
CATTI 证书编号：201906009370001040
组织机构：易传思达（北京）信息技术有限公司
电话：010-60537024　邮箱：3004146455@qq.com
组织地址：北京市平谷区中关村科技园平谷园 6 区
签名：季相娣　翻译日期：2025 年 2 月 6 日

3. Interview Questions Tables

3.1 CPP Interview Questions Table for Focus group (Item 7 did not meet the acceptable IOC threshold and was removed in Interview)

CPP Dimensions	Definitions	Focus on the Dimensions	Interview Questions	Source Adapt from
**Character Analysis**	Character Analysis is the process of examining a character's motivations, relationships, and psychological depth to create a cohesive and believable portrayal Grounded in both acting pedagogy and psychological frameworks, it integrates textual deconstruction and emotional exploration to enhance narrative coherence and authenticity (Pugh et al., 2013; Brownell, 2010; Iorga, 2023).	To explore how students develop their understanding of their character's motivations and relationships in *The Last Empress and Der Ling* and to evaluate the role of Gestalt techniques in enhancing this process.	1. What approach did you use to analyze your character in *The Last Empress and Der Ling*?<br>2. How did the Empty Chair Technique help you understand your character's emotions and decision-making process?	((Herman, 2007; Pascual-Leone and Baher, 2023)
**Communication Skills**	Communication Skills in acting encompass the ability to convey emotions, intentions, and character depth through verbal articulation, vocal modulation, and non-verbal expressions such as gestures and body language. Effective communication ensures coherence between dialogue and physicality, enhancing audience engagement and character authenticity (Nicole, 2019; Holmström et al., 2024).	To investigate how students effectively use verbal and non-verbal cues for character portrayal in their assigned scenes and how Dialogue Exercises enhance these abilities.	3. Which dialogue exercises were most effective in improving your verbal and non-verbal communication skills?<br>4. In what ways did the Exaggeration Technique help you enhance your physical expressiveness on stage?	(Novotny, Frank and Grizzard, 2021; Cheng *et al.*, 2019)
**Relational Dynamics**	Relational Dynamics refers to the way characters interact, communicate, and influence each other within a performance, shaping the authenticity and emotional depth of their relationships. This process involves understanding power structures, emotional exchanges, and non-verbal cues to create believable and dynamic character interactions (Andrášik et al., 2024; Siegel, 2021; Konijn, 2000).	To assess how students interpret and portray relationships between characters, such as Der Ling and Empress Dowager Cixi, and how Role Play enhances relational understanding.	5. How did role play contribute to building believable interactions between your character and others?<br>6. How did Gestalt techniques influence your ability to portray relationships between characters in the script?	(Garrett, 2024; Tan, 2018)
**Internal and External Performance Design**	Internal performance design focuses on the actor's internal processes, such as emotions and motivations, while external performance design involves the physical and interactive aspects of performance. These concepts are crucial in creating a holistic and authentic portrayal of characters in various performance settings (Demaree et al., 2004; Panero, 2021; Berry et al., 2022).	To explore how students balance emotional depth and physical expression, particularly in portraying complex characters like Empress Dowager Cixi, using techniques like Exaggeration.	7. How did you integrate emotional depth with physical movement in your portrayal of (your character)?<br>8. Can you describe how Gestalt-based methods helped you externalize internal emotions through body language during performance?<br>9. How did these techniques affect your performance in specific moments, such as scenes involving intense conflict or inner monologue?	(Cuffari, 2012; Hague, 2016)
**Stage Presence**	Stage Presence is the actor's ability to command attention, maintain audience engagement, use text-based theatre rehearsals and workshops, and convey authenticity through emotional connection, physical expressiveness, and spatial awareness. It involves a dynamic interplay of confidence, focus, and responsiveness, ensuring a compelling and immersive performance (Goldstein, 2009; Mann, 2020; Mirodan, 2017).	To evaluate how students sustain audience engagement and develop stage presence, focusing on how Gestalt interventions like Role Play enhance confidence and emotional resonance.	10. During emotionally heightened or climactic scenes, which methods helped you achieve a greater sense of authenticity?<br>11. Compared to traditional methods, did Gestalt techniques boost your confidence as a performer? How did they affect your ability to command the stage?	(Lavan, Scott and McGettigan, 2016; Capra, Berthaut and Grisoni, 2020)

3.2 Expert Semi-Structured Interview Table

Interview Focus Areas	Focus on the Dimensions	Interview Questions	Source Adapt From
**Evaluation of CPP Dimensions**	To explore how Gestalt therapeutic techniques specifically impacted each of the five CPP dimensions: Character Analysis, Relational Dynamics, Communication Skills, Internal and External Performance Design, and Stage Presence (Konijn, 2000; Panero & Winner, 2021).	1. From your professional perspective, how do Gestalt therapeutic techniques (GTT) influence students' performance across the various dimensions of Character Portrayal Proficiency (CPP)?<br>2. How would you assess the extent to which GTT contributes to students' understanding of character development and interpersonal relationships on stage?	(Day, 2016; Lerner and Schmid Callina, 2014)
**Effectiveness of GTT in Acting Training**	To evaluate the perceived strengths and limitations of using GTT (Empty Chair Technique, Role Play, Dialogue Exercises, and Exaggeration Technique) in developing acting skills (Brownell, 2010).	3. What is your opinion on the dialogue-based exercises within GTT? In your view, what aspects of these exercises are most effective in improving students' verbal and non-verbal communication?<br>4. In what ways do you see the GTT exaggeration techniques differing from Stanislavsky's action methods? What specific advantages, if any, do they offer in actor training?	(Liu, Calvo and Lim, 2016; Żywiczyński *et al.*, 2024)
**Recommendations for Future Application**	To gather suggestions for refining the use of GTT in acting pedagogy and for improving the assessment of CPP (Street, 2006; Holmström et al., 2024).	5. Do you believe the role play component of GTT provides a more effective pathway for students to build believable character interactions compared to traditional methods? Why or why not?<br>6. When comparing internal and external performance design between the experimental and control groups, what notable differences or similarities did you observe? Were there any specific strengths or areas for improvement?	(Stokoe, 2014; Möller and Köller, 2001)
**Challenges Observed in the Experiment**	To understand any difficulties faced by students and instructors during the experimental process (Siegel, 2021).	7. In scenes requiring emotional intensity or climax, did you find that students in the experimental group demonstrated a stronger sense of authenticity on stage? Please elaborate.<br>8. Based on your observations, did the application of GTT appear to enhance students' confidence and their ability to maintain stage presence during performance?	(Lenton *et al.*, 2013; Pelton, 2014)
**Insights on Student Learning and Development**	To analyze the long-term impact of GTT on students' engagement, learning, and performance development (Panero, 2021).	9. What considerations should educators keep in mind when incorporating GTT into acting training? What potential challenges do you foresee in its wider implementation?<br>10. Do you think GTT holds long-term value for acting education? In your view, what directions should future research take to explore its full potential?	((Robson, Krolikowska and Williams, 2025; McNaughton, 2010)

frontiers

4. Ethics Documents

4.1 The AU Institutional Review Board

มหาวิทยาลัยอัสสัมชัญ
ASSUMPTION UNIVERSITY

Assumption University

Certification

No. 10/2025

This document is issued to certify that

Research Title: "Exploring the Integration of Gestalt Therapeutic Tchniques into Character Portrayal Proficiency in Undergraduate Acting Students".

Researcher Name: Ms. Dian Li

The acquired documents for consideration are as follows:

1. Research Project
2. Research participations' Information
3. Research Consent Form
4. Research Questionnaire

All mentioned documents have been approved for the Exemption Review by the AU Institutional Review Board.

Issued on: April 18, 2025

(Assoc.Prof.Dr. Chanintorn Jittawiriyanukoon)
Chairman of the AU Institutional Review Board

ADDRESS: ABAC, ASSUMPTION UNIVERSITY, HUA MAK, BANGKOK 10240. TEL. 0-2783-2222 http://www.au.edu

4.2 Bilingual Consent Forms

INFORMED CONSENT FORM FOR EXPERT PARTICIPANTS
专家参与者知情同意书

RESEARCH STUDY TITLE / 研究课题标题:

The Impact of Gestalt Therapeutic Techniques on Character Portrayal Proficiency in Undergraduate Acting Students

格式塔治疗技术对本科表演学生角色塑造能力的影响

Principal Investigator / 主要研究员: Dian Li

Program / 所属项目: Doctor of Philosophy in Arts, Music, Sport and Entertainment Management

Institution / 所属机构: Assumption University of Thailand

Dear Expert, / **尊敬的专家:**

You are invited to participate in a research study that explores how Gestalt therapeutic techniques (GTT) may enhance character portrayal in acting education. Your professional insight will help evaluate the practical and pedagogical value of these techniques. / 诚邀您参与本研究。本研究旨在探讨格式塔治疗技术（GTT）如何有助于提升表演教育中的角色塑造能力。您的专业见解将有助于评估这些技术在教学实践中的应用价值。

Purpose of the Study:

This study aims to assess the integration of selected Gestalt therapeutic methods including the Empty Chair, Dialogue Exercises, Role Play, and Exaggeration within undergraduate acting programs, focusing on their impact on Character Portrayal Proficiency (CPP). /

研究目的: 本研究旨在评估将若干格式塔治疗方法（如空椅子技术、对话练习、角色扮演和夸张技巧）融入本科表演课程中的效果，重点关注其对角色塑造能力（CPP）的影响。

Participation:

As a participant, you will be asked to (1) assess the pre-test and post-test performance of two student classes using a CPP rubric, and (2) join a semi-structured interview lasting

approximately 30–45 minutes, conducted either in person or online. With your consent, the interview will be audio-recorded and transcribed for academic analysis. /
参与内容:作为参与者，您将被邀请：（1）使用 CPP 评分标准对两个学生班级的前测和后测表演进行评估；（2）参与一次时长约 30 - 45 分钟的半结构式访谈（可线上或线下进行）。在征得您的同意后，访谈将被录音并用于学术分析。

Confidentiality:
All collected information will be kept strictly confidential and used solely for academic purposes. No personally identifiable information will appear in any publication or presentation resulting from this study. Your identity will remain anonymous in all reports. /
保密条款:所有收集到的信息将严格保密，仅用于学术研究目的。本研究的任何出版物或展示中均不会出现可识别您身份的信息，您的身份在所有报告中将保持匿名。

Benefits and Risks:
While there are no direct benefits or risks, your contribution may support improvements in acting pedagogy and interdisciplinary teaching approaches. /
潜在益处与风险:本研究没有已知的直接利益或风险，但您的参与将有助于推动表演教学法和跨学科教学方法的发展。

CONSENT AND SIGNATURE / 同意与签字

I have read and understood the above information. I voluntarily agree to participate in this study. /
本人已阅读并理解上述内容，自愿同意参与本研究。

Signature of Participant / 参与者签名: ____

Date / 日期: ______________________

Investigator Signature / 研究员签名: ___________________________________

Date / 日期: ______________________

Authorized by / 授权人

指导教师

Signature / 签名: ____

Date / 日期: ______________________

I, Ji Xiangdi, translator of E-TRANSTAR (Beijing) Information Technology Co., Ltd., confirm this is a true and accurate translation of the original document.

CATTI Certificate No.: 201906009370001040 Organization: E-TRANSTAR (Beijing) Information Technology Co., Ltd.

Tel: 010-60537024 Email: 3004146455@qq.com

Organization Address: 201-212057, Zone 6, Pinggu Park, Zhongguancun Science and Technology Park, Pinggu District, Beijing

Signature: Ji Xiangdi Date of Translation: January 22, 2025

E-Transtar (Beijing) Information Technology Co., Ltd. 易思达（北京）信息技术有限公司 翻译专用章 Valid for Translation Only

INFORMED CONSENT FORM FOR STUDENT PARTICIPANTS
学生参与者知情同意书

RESEARCH STUDY TITLE / 研究课题标题:
The Impact of Gestalt Therapeutic Techniques on Character Portrayal Proficiency in Undergraduate Acting Students
格式塔治疗技术对本科表演学生角色塑造能力的影响

Principal Investigator / 主要研究员: Dian Li
Program / 所属项目: Doctor of Philosophy in Arts, Music, Sport and Entertainment Management
Institution / 所属机构: Assumption University of Thailand

Dear Student Participant, / **亲爱的学生参与者：**

You are invited to voluntarily participate in a research study that investigates the use of Gestalt therapeutic techniques (GTT) in undergraduate acting training. This study aims to explore how selected GTT methods may improve your character portrayal abilities and overall performance skills. / 您被邀请自愿参加一项关于将格式塔治疗技术（GTT）应用于本科表演训练的研究。本研究旨在探索选定的 GTT 方法是否能提升您的角色塑造能力及整体表演水平。

Purpose of the Study: This study aims to assess the integration of selected Gestalt therapeutic methods including the Empty Chair, Dialogue Exercises, Role Play, and Exaggeration within undergraduate acting programs, focusing on their impact on Character Portrayal Proficiency (CPP). /
研究目的: 本研究旨在评估将若干格式塔治疗方法（如空椅子技术、对话练习、角色扮演和夸张技巧）融入本科表演课程中的效果，重点关注其对角色塑造能力（CPP）的影响。

Participation: Your participation will span the full 16-week training period. /
参与内容: 您的参与将覆盖整个 16 周的训练周期。

In Weeks 1 and 16, you will participate in a pre-test and post-test performance assessment. / 第 1 周和第 16 周，您将参加前测与后测的表演评估；

In Weeks 2–15, you will attend regular acting classes incorporating selected Gestalt therapeutic techniques. These sessions will include practical exercises, scene rehearsals, and group discussions. / 第 2 至第 15 周，您将参与定期的表演课程，课程中将融入格式塔治疗技术。这些课程包括实践练习、剧目排练与小组讨论；

In Week 16, approximately eight students from the experimental group will be randomly selected to participate in a focus group interview, lasting about 60–75 minutes. This interview will explore your personal experiences and reflections on the application of GTT in your acting process. Interviews will be audio-recorded with your consent. / 第 16 周，实验组将随机选取大约 8 名学生参与一次焦点小组访谈，访谈时长约为 60 – 75 分钟，内容围绕您在表演过程中使用 GTT 的个人体验与反思。所有访谈将在征得您同意后进行录音。

Confidentiality: All responses and personal data will remain strictly confidential. Your identity will be anonymized in all reports and publications. Participation or non-participation will not affect your academic standing. /
保密条款:所有收集到的信息将严格保密，仅用于学术研究目的。您的身份将在所有报告与出版物中匿名处理，您的参与或不参与不会影响您的学术成绩或学术地位。

Benefits and Risks: There are no known risks associated with participation. This study may help you gain deeper insight into your acting process and provide innovative tools for character development. /
潜在益处与风险:本研究不存在已知的风险。研究可能有助于您更深入了解自己的表演过程，并为角色发展提供创新方法与工具。

CONSENT AND SIGNATURE / 同意与签字

（I have read and understood the above information. I voluntarily agree to participate in this study.）
本人已阅读并理解上述内容，自愿同意参与本研究。

Signature of Participant / 参与者签名: ____

Date / 日期: ____________________

Investigator Signature / 研究员签名: ________________________________

Date / 日期: ____________________

Authorized by / 授权人

指导教师

Signature / 签名: ______________________________________

Date / 日期: ____________________

I, Ji Xiangdi, translator of E-TRANSTAR (Beijing) Information Technology Co., Ltd., confirm this is a true and accurate translation of the original document.

CATTI Certificate No.: 201906009370001040 Organization: E-TRANSTAR (Beijing) Information Technology Co., Ltd.

Tel: 010-60537024 Email: 3004146455@qq.com

Organization Address: 201-212057, Zone 6, Pinggu Park, Zhongguancun Science and Technology Park, Pinggu District, Beijing

Signature: Ji Xiang di Date of Translation: January 22, 2025

PHOTOGRAPH USE IN PUBLICATION – ADDITIONAL CONSENT FORM / 照片使用知情同意书（用于学术发表）

RESEARCH STUDY TITLE / 研究课题标题：
The Impact of Gestalt Therapeutic Techniques on Character Portrayal Proficiency in Undergraduate Acting Students
格式塔治疗技术对本科表演学生角色塑造能力的影响
Principal Investigator / 主要研究员： Dian Li；Changhan Li; Lu Zhu
Program / 所属项目： Doctor of Philosophy in Arts, Music, Sport and Entertainment Management
Institution / 所属机构： Assumption University of Thailand

Dear Student Participant, / 亲爱的学生参与者：

During this study, photographs may be taken during class sessions, rehearsals, and performances to visually document the teaching methods and exercises discussed in the research. These images may be included in academic publications (including journal articles, conference presentations, and educational materials). This separate consent form is to obtain your explicit permission for the use of such photographs.

在本研究进行期间，课堂练习、排练及演出过程中可能会拍摄照片，用于直观记录本研究讨论的教学方法与练习。这些图像可能会被收录在学术出版物中（包括期刊文章、学术会议报告及教育材料）。本独立知情同意书旨在就此类照片的使用征得您的明确许可。

Purpose of Photographs / 照片用途：

Photographs will be used solely for academic and educational purposes, including:

- Publication in scientific journals (e.g., Frontiers in Psychology)
- Academic conference presentations
- Educational lectures and teaching materials
- University research archives

照片将仅用于学术和教育目的，包括：

- 科学期刊发表（如《心理学前沿》）
- 学术会议报告
- 教育讲座与教学材料
- 大学研究档案

Your Rights / 您的权利:

- Your consent for photograph use is voluntary and separate from your participation in the main study
- You may choose not to consent to photographs being used, and this will not affect your academic standing or participation in the study
- You may withdraw your consent at any time before publication by contacting the researcher
- Once the article is published, photographs cannot be removed from the published version
- 您对照片使用的同意是自愿的，且独立于您参与主要研究的同意
- 您可以选择不同意照片被使用，这不影响您的学术成绩或研究参与
- 您可以在文章发表前随时联系研究者撤回同意
- 文章一旦发表，已出版版本中的照片将无法移除

Photograph Details / 照片说明:

- Photographs may show your face and other identifiable features
- No names will be directly associated with photographs in publications
- Images will be published open access and will be permanently available online
- Photographs will not be used for commercial purposes
- 照片中可能显示您的面部及其他可识别特征
- 发表时不会将姓名与照片直接关联
- 图像将以开放获取形式发表，永久在线可见
- 照片不会用于商业目的

CONSENT AND SIGNATURE / 同意与签字

I have read and understood the above information regarding the use of photographs in academic publications. I voluntarily agree to allow photographs taken of me during this study to be used as described above.
本人已阅读并理解上述关于照片在学术出版物中使用的说明。我自愿同意本研究中拍摄的我的照片按上述说明使用。

Please check one option / 请选择一项:

□ **I CONSENT to the use of my photographs in publications**
□ **我同意我的照片在出版物中使用**

□ **I DO NOT CONSENT to the use of my photographs in publications**
□ **我不同意我的照片在出版物中使用**

Participant Name / 参与者姓名: ______________________________

Signature / 签名: ______________________________

Date / 日期: __________________

Investigator Signature / 研究员签名: ______________________________

Date / 日期: __________________

Authorized by / 授权人

Signature / 签名: ______________________________

Date / 日期: __________________

This form will be kept separately from study data and stored securely in a locked file accessible only to the research team. /
本表格将与研究数据分开保存，并妥善存放于上锁的文件柜中，仅研究团队可访问。

I, Ji Xiangdi, translator of E-TRANSTAR (Beijing) Information Technology Co., Ltd., confirm this is a true and accurate translation of the original document.

CATTI Certificate No.: 201906009370001040 Organization: E-TRANSTAR (Beijing) Information Technology Co., Ltd.

Tel: 010-60537024 Email: 3004146455@qq.com

Organization Address: 201-212057, Zone 6, Pinggu Park, Zhongguancun Science and Technology Park, Pinggu District, Beijing

Signature: Ji Xiang di Date of Translation: January 22, 2025

翻译专用章
Valid for Translation Only
